# STK-mediated FadR phosphorylation regulates the acid resistance and virulence of *Streptococcus suis*

**DOI:** 10.1371/journal.ppat.1013534

**Published:** 2025-09-25

**Authors:** Sen Li, Zhe Ma, Huixing Lin, Fei Pan, Hong Zhou, Jinsheng Tang, Hongjie Fan

**Affiliations:** 1 MOE Joint International Research Laboratory of Animal Health and Food Safety, College of Veterinary Medicine, Nanjing Agricultural University, Nanjing, China; 2 Jiangsu Co-innovation Center for Prevention and Control of Important Animal Infectious Diseases and Zoonoses, Yangzhou University, Yangzhou, China; 3 College of Animal Science, Anhui Science and Technology University, Fengyang, China; Murdoch Children's Research Institute, AUSTRALIA

## Abstract

The phagolysosomes of macrophages play a crucial role in eradicating pathogenic microorganisms, but bacteria have evolved sophisticated mechanisms to survive in the acidic environment of phagolysosomes, leading to host infection and subsequent dissemination. However, it is largely unknown how bacteria sense the extracellular stimuli and regulate their acid tolerance capacity to resist the killing by host immune cells. Here, we report the new substrate FadR of the serine/threonine kinase (STK) in *Streptococcus suis* serotype 2 (SS2) and demonstrate that the phosphorylation site is Thr230. Notably, FadR phosphorylation significantly enhances the acid resistance of SS2, leading to an increase in the lethality of SS2 in mice, and a marked increase in bacterial load in the blood and various organs, and more severe pathological changes in various organs of the mice. Interestingly, this study further indicated that FadR protein can bind to the promoter of arginine deiminase (*adi*), and FadR phosphorylation enhances its binding ability to the *adi* promoter and increases *adi* transcription levels. The increase of ADI in SS2 promotes the metabolism of arginine and increases the ammonia content, thus enhancing the acid resistance and intracellular survival capacity of the bacteria in macrophages. Altogether, the research reveals an acid resistance regulatory mechanism that bacteria can utilize the STK-FadR signaling axis to sense changes in the external acidic environment, and then manipulate the ADI system to enhance bacterial resistance to acidic environment or host immunity.

## Author summary

The serine/threonine kinase (STK) is involved in multiple important biological response pathways and is closely related to the pathogenicity of bacteria. However, the acid tolerance mechanism of STK in SS2 is still unclear. These findings suggests that STK can increase the transcription and expression levels of the arginine deiminase (*adi*) gene by phosphorylating the transcription factor FadR. Importantly, the increase of ADI in SS2 leads to excessive ammonia production under acidic stimulation, thereby maintaining the stability of the intracellular pH and improving acid resistance and intracellular survival ability in macrophages. This study further expands the phosphorylation regulatory network of pathogens and provides new strategies for the prevention and control of pathogens.

## Introduction

In the early stages of infection, the innate immune system is critical for limiting microbial survival and spread. Macrophages play a central role in innate immunity and exert a special function in phagocytosing various pathogenic microorganisms within the body [1]. However, bacteria have evolved sophisticated and efficient ways to overcome these innate immune system [2]. The capsule can resist phagocytosis, transcription factors OxyR, SoxR, and SoxS are involved in oxidative stress responses, and the periplasmic protease MarP enhances acid tolerance [2–4]. However, it is still unclear how bacteria perceive extracellular stimuli and enhance their resistance to macrophage killing, thereby evading innate immunity.

Macrophages eliminate pathogens by creating an acidic environment, which is an important strategy for the host to defend against the invasion of foreign pathogens. However, the acid resistance of pathogens significantly increases their chances of survival within macrophages [5]. These pathogens can resist acidic environments through various mechanisms, such as proton depletion, ammonia production, the regulation of their own membrane structure, and chemotaxis [6–8]. HdeA and HdeB form a unique periplasmic space for gram-negative bacteria, endowing them with acid resistance [9]. The bacterial stress resistance components, such as the CiaRH two-component system, enhance their growth in acidic environments, indicating that bacteria may have the ability to perceive changes in the external environment to cope with acidic environments and intracellular survival [10].

Producing ammonia to resist acidic environments and enhance their survival ability is an important acid resistant pathway for bacteria [7,11]. The arginine deiminase system, comprising arginine deiminase (*adi*), ornithine carbamoyltransferase, and carbamate kinase, catalyzes the irreversible hydrolysis of L-arginine into L-citrulline and ammonia, playing a crucial role in regulating the intracellular acid‒base balance in bacteria [12–14]. However, it remains unknown whether bacteria can sense changes in the external environment and manipulate this system to enhance their acid tolerance.

For bacteria, sensing extracellular stimuli to cope with adverse environmental changes is crucial for their survival. The serine/threonine kinase (STK) is an important marker of bacterial phosphorylation signal transduction, and in addition to autophosphorylation, it can also catalyze the phosphorylation of specific substrates, thus initiating bacterial self-protection mechanisms to cope with adverse environments [15]. STK regulates not only bacterial growth and cell division but also plays a role in antibiotic persistence, virulence, infection, metabolism, chromosomal biology, and cell differentiation [15,16]. STK can regulate the transcriptional activity of target genes through phosphorylating modifications of transcription factors, as well as modulate the activity of enzyme protein substrates [17–21]. *Streptococcus suis* serotype 2 (SS2) is an important zoonotic pathogen that can cause meningitis and septicemia in pigs, and meningitis and streptococcal toxic shock-like syndrome (STSLS) in humans [22,23]. In recent research, we reported that SS2 can regulate its antioxidant capacity and capsule synthesis through STK [24,25]. Furthermore, a previous study has shown that STK is involved in the acid stress response of bacteria [26]. However, the mechanism by which STK regulates bacterial acid tolerance to enhance bacterial survival in macrophages remains to be further investigated.

In this study, we demonstrated for the first time that FadR transcription factor and its phosphorylation play critical roles in the acid resistance regulatory mechanism of SS2. FadR belongs to the GntR family, consisting of an N-terminal DNA binding domain and a C-terminal effector binding domain [27]. We demonstrated that FadR can be specifically phosphorylated by STK, and the STK-FadR axis is active. Interestingly, the FadR phosphorylation modification occurs at the Thr230 residue in its C-terminal effector-binding domain. Importantly, FadR is able to bind the promoter region of *adi*, and FadR phosphorylation enhances its binding ability to the *adi* promoter and expression levels of ADI protein, accelerating the arginine-to-ammonia conversion process, thereby significantly increasing the acid resistance and virulence of SS2. Altogether, our research reveals an acid resistance regulatory mechanism that SS2 can operate ADI system to effectively control the intracellular acid‒base balance using the STK-FadR axis, which providing new insights into the pathogenic mechanism of SS2 and offers a reference for exploring corresponding vaccine and drug targets.

## Results

### Thr-phosphorylation of FadR protein is specifically mediated by STK

**Fig 1:**
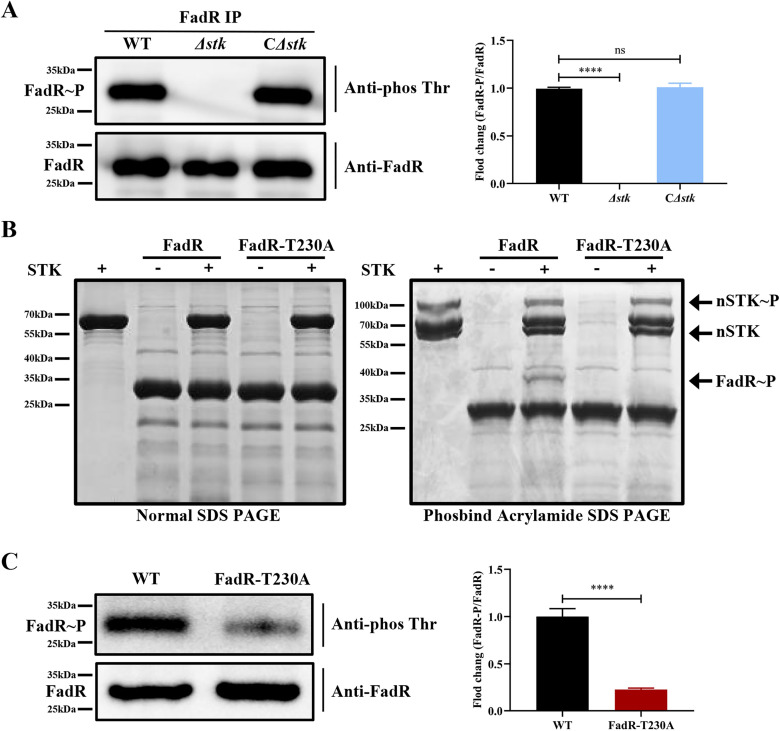
FadR is phosphorylated by STK. **(A)** FadR polyclonal antibody was used to perform immunoprecipitation (IP) experiments with whole proteins of WT SS2, *Δ*stk**, and C*Δ*stk** strains. The pulled-down protein was separated by SDS‒PAGE, and Western blot analysis was performed using anti-phosphorylated Thr antibody and FadR polyclonal antibody at a 1:1000 dilution. The band intensity was analyzed relative to the FadR protein phosphorylation level in WT SS2 strain. **(B)** The purified GST nSTK (15 μg) and substrate protein (5 μg) were mixed in phosphorylation buffer and incubated at 37°C for 2 h, and a phosphorylation reaction was performed. The samples were subjected to 12% conventional SDS‒PAGE (left) and Phosbind acrylamide SDS‒PAGE (right). The separated proteins were observed by Coomassie Brilliant Blue staining. **(C)** The IP experiments of WT SS2 and FadR-T230A whole proteins were performed separately with FadR polyclonal antibodies. The pull-down protein was detected by the method described in **(A)**. The band intensity was analyzed relative to the FadR protein phosphorylation level in WT SS2 strain. All data are representative of at least three independent experiments with similar results. The data were statistically analyzed by One-way ANOVA **(**A**)** and an unpaired *t*-test ****(C**)**. ^ns^, **P* *> 0.05; ^****^, *P* < 0.0001.

The SS2 genome encodes a *stk* gene, which includes the typical N-terminal kinase domain in the cytoplasm and the C-terminal PASTA domain in the cytoplasm, separated by a transmembrane region [25]. STK has been proven to be involved in many crucial physiological processes of bacteria [15,19,28]. The global phosphorylation profiles of wild-type (WT) SS2 and *Δ*stk** strains were compared through high-throughput phosphoproteomics and protein quantitative omics. According to the phosphoproteomic results, a phospho-peptide signal of FadR (ZY05719_05575) in *Δ*stk** strain was significantly attenuated (S1 Table) compared with that of WT SS2 strain, and the mass spectrometry results revealed that Thr230 was a potential phosphorylation site (S1 Fig). Next, we further investigated whether STK can phosphorylate FadR *in vivo*. Immunoprecipitation (IP) analysis revealed that compared with WT strain, the phosphorylation of FadR disappeared in *Δ*stk** strain, while there was no difference in the phosphorylation level of FadR in C*Δ*stk** strain (Fig 1A), which is consistent with the results obtained from mass spectrometry analysis. We subsequently attempted to determine the phosphorylation sites mediated by STK in FadR. The recombinant FadR protein and the non-phosphorylatable FadR-T230A mutant protein were expressed, purified, and incubated with nSTK *in vitro*. Phos-tag SDS‒PAGE assay was used to determine the phosphorylation level of FadR, and the results revealed that only FadR protein presented a shifted band (Fig 1B), which also indicated that only T230 at the C-terminal of FadR was phosphorylated by STK. In addition, the IP analysis reveals that the phosphorylation level of FadR in FadR-T230A strain was significantly lower than that in WT SS2 (Fig 1C). Altogether, these results indicated that Thr230 in FadR is the only target site for STK.

### FadR phosphorylation in SS2 is regulated by extracellular stimuli

**Fig 2:**
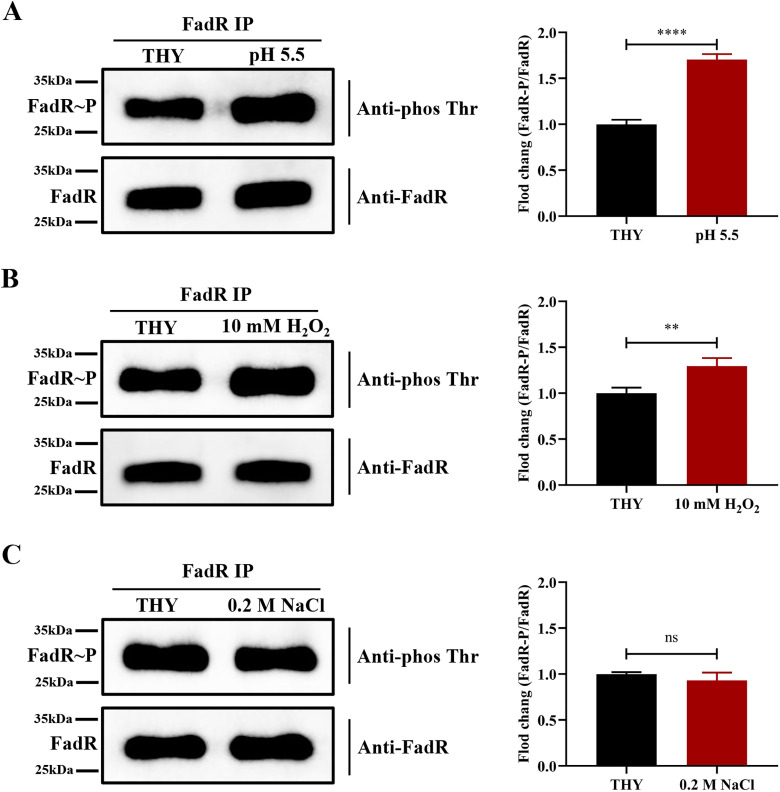
Increased phosphorylation level of FadR in acidic environments. **(A)** Exponentially growing cells were treated with pH 5.5 THY adjusted with hydrochloric acid for 30 min. **(B)** Exponentially growing cells were treated with THY supplemented with 10 mM H_2_O_2_ for 30 min. **(C)** Exponentially growing cells were treated with THY supplemented with 0.2 M NaCl for 30 min. **(A-C)** Immunoprecipitation (IP) experiments of all WT SS2 whole proteins were performed separately with FadR polyclonal antibodies. The pull-down protein was detected by the method described in Fig 1A. Bar graphs showed the percentage of phosphorylated and total FadR in different treatment groups. All experiments were repeated three times. ^ns^, **P* *> 0.05; ^**^, *P* < 0.01; ^****^, *P* < 0.0001 (an unpaired *t*-test).

The level of STK-mediated phosphorylation of substrate proteins in bacteria may be influenced by the external environment [25]. To explore the important physiological significance of FadR phosphorylation in the life process of SS2, we subjected WT SS2 strains to various environmental stresses and evaluated the *in vivo* phosphorylation level of FadR. To avoid the possibility that the observed differences in FadR phosphorylation states might be due to the degree of bacterial death rather than the nature of the applied stress, we conducted experiments using weak acid (THY, pH 5.5), low concentration hydrogen peroxide (THY, 10 mM H_2_O_2_), and low-salt (THY, 0.2 M NaCl) stress for a 30 min experiment. Additionally, we also tested the bacterial viability before and after the stress, and results showed that these environmental stresses had no significant effect on bacterial survival (S2 Fig). The results revealed that FadR phosphorylation levels significantly increased in acidic environments and hydrogen peroxide, while osmotic pressure had no effect on FadR phosphorylation (Fig 2A–2C). Therefore, we speculated that the STK-FadR axis may play an important role in the acid stress response of SS2.

### Phosphorylation of FadR enhances SS2 virulence

STK-mediated phosphorylation of substrates had a significant impact on the pathogenicity and virulence of bacteria [24,25]. To investigate the effect of STK-mediated FadR phosphorylation on the virulence of SS2, the growth curves of WT SS2 strain, *fadR*-deleted strain *Δ*fadR** (S3A Fig), Complementation strain C*Δ*fadR** (S3B Fig), phospho-ablative strain FadR-T230A (mimics non-phosphorylated Thr residues, which alanine replaces threonine), and phosphomimetic strain FadR-T230E (mimics phosphorylated Thr residues) (S3C Fig) were first measured under the same conditions. The results showed that these mutant strains had no difference in growth rate compared to WT SS2 strain (S3D Fig). Furthermore, compared with WT SS2 strain, there were no significant differences in the transcription and expression levels of FadR in C*Δ*fadR** strains (S4A and S4B Fig).

**Fig 3:**
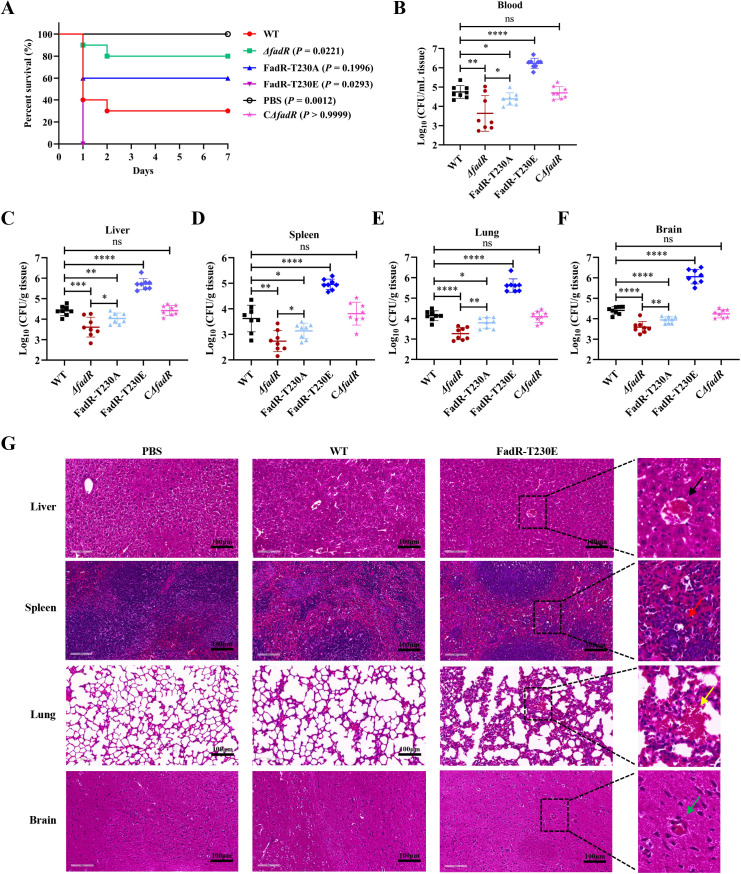
Phosphorylation of FadR enhances bacterial virulence. **(A)** BALB/c mice were intraperitoneally injected with WT SS2, *Δ*fadR**, FadR-T230A, FadR-T230E, and C*Δ*fadR** strains at a dose of 2 × 10^8^ CFUs/mice (n = 10 mice/group), and survival time was closely monitored. **(B-F)** After the intraperitoneal injection of 2 × 10^7^ CFUs of WT SS2, *Δ*fadR**, FadR-T230A, FadR-T230E, and C*Δ*fadR** strains into BALB/c mice, the bacterial loads in the blood **(B)**, liver **(C)**, spleen **(D)**, lungs **(E)**, and brain **(F)** were measured at 24 hpi (n = 8 mice/group). **(G)** H&E staining of lung and spleen tissue sections at 24 hpi from mice inoculated with PBS, WT SS2, and FadR-T230E strains by intraperitoneal injection. In the enlarged area of the images (black dashed frame), the black arrow indicates hepatic steatosis, with varying numbers of small circular vacuoles in the cytoplasm, and many congested blood vessels and hepatic sinusoids. The red arrow indicates a decreased number of lymphocytes. The yellow arrow indicates alveolar congestion, thickening of alveolar walls, and alveolar collapse. The blue arrow indicates glial cell nodules, and some bruising had appeared. Scale bar, 100 μm. The data were statistically analyzed by the Log-rank test **(A)** and One-way ANOVA **(B-F)**. ^ns^, *P >* 0.05; ^*^, *P* < 0.05; ^**^, *P* < 0.01; ^***^, *P* < 0.001; ^****^, *P* < 0.0001.

To explore the effect of FadR and its phosphorylation on the virulence of SS2, an animal intraperitoneal infection challenge test was performed. Animal experimental model revealed that, compared with that of the mice injected with WT SS2 strain (30%), the survival rate of the mice injected with *Δ*fadR** (80%) and FadR-T230A (60%) strains were significantly increased, the survival rate of the mice injected with FadR-T230E (0%) strain was significantly decreased, and the survival rate of the mice injected with C*Δ*fadR** (30%) strain showed no significant change (Fig 3A). In addition, to investigate the role of FadR in *in vivo* infection of SS2, we compared the colonization efficiency of WT SS2 and mutant strains in BALB/c mice. The results indicated that, compared with WT SS2 strain, the CFUs of blood and various organs in the mice infected with *Δ*fadR** and FadR-T230A strains were significantly decreased, the CFUs of blood and various organs in the mice infected with FadR-T230E strain were significantly increased, and the CFUs of blood and various organs in the mice infected with C*Δ*fadR** strain showed no significant change (Fig 3B–3F). Meanwhile, compared with *Δ*fadR** strain, the CFUs of blood and various organs in the mice infected with FadR-T230A strains were significantly increased (Fig 3B–3F). Notably, the results revealed that FadR and its phosphorylation significantly weakened the ability of tissues/organs in infected mice to clear pathogenic microorganisms.

*Streptococcus suis* can usually cause meningitis, septicemia, endocarditis, and arthritis in pigs, with a high mortality rate [29]. Next, the histopathological analysis of the liver, spleen, lung, and brain tissues from mice challenged with SS2 revealed hepatic steatosis, with varying numbers of small circular vacuoles in the cytoplasm, and many blood vessels and hepatic sinusoids were congested; the number of lymphocytes in the spleen decreased; alveolar congestion, thickening of alveolar walls, and alveolar collapse were observed; glial cell nodules and some bruising appeared in the brain (Fig 3G). Compared with WT SS2 strain, the liver, spleen, lung and brain tissues of the mice infected with FadR-T230E presented more severe pathological manifestations (S5A–5D Fig). Altogether, these animal experiments indicated that FadR is indispensable for whole virulence and pathogenicity of SS2, and phosphorylation plays an important role in FadR functional process.

### FadR binds to promoter regions of the *adi*

**Fig 4:**
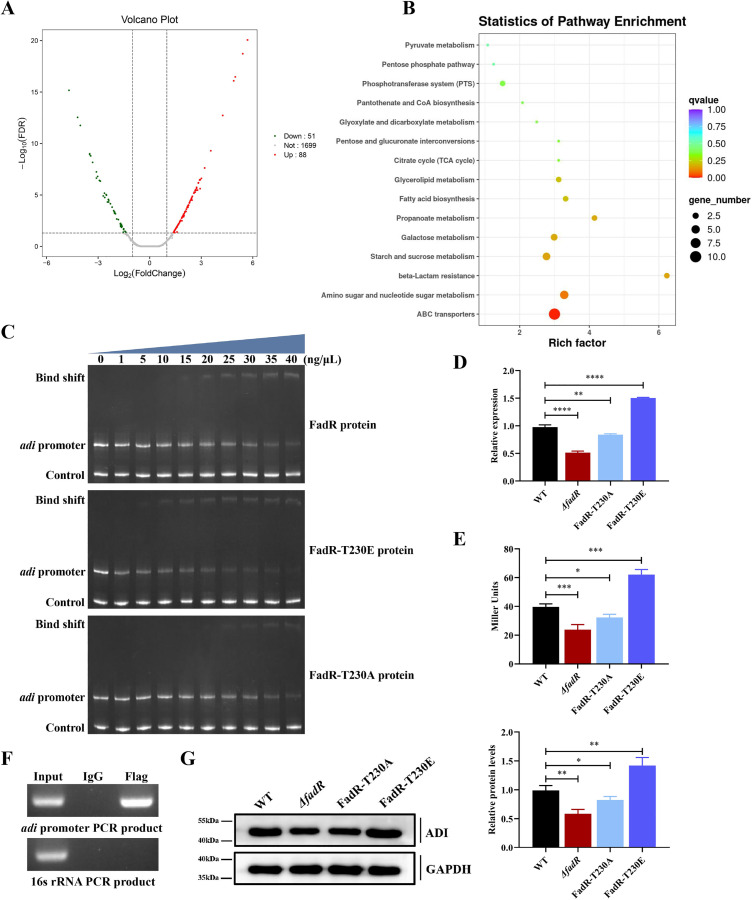
Phosphorylation of FadR increases the transcription of *adi.* **(A)** Volcano plot showing the differential gene expression of the RNA-seq results between WT SS2 and *Δ*fadR** strains. The genes that were significantly upregulated or downregulated by at least 2-fold are represented by red and green dots, respectively. **(B)** The statistical analysis of KEGG pathway enrichment is represented by scatter plots of the differentially expressed genes. **(C)** FadR, FadR-T230A, and FadR-T230E proteins were incubated with DNA fragments (80 ng) ~300 bp in length from the *adi* promoter region. The negative control was a DNA fragments (80 ng) ~197 bp in length from the *enolase* promoter region. The reaction was carried out at 37°C for 30 min, separated with a 6% polyacrylamide gel in 0.5 × TBE buffer, and stained with ethidium bromide for imaging. **(D)** The *adi* transcript levels in WT SS2, *Δ*fadR**, FadR-T230A, and FadR-T230E strains were determined by RT‒qPCR. **(E)** The pTCV-*lacZ* recombinant plasmid containing the *adi* promoter sequence was electroporated into the WT SS2, *Δ*fadR**, FadR-T230A, and FadR-T230E strains, and *adi* promoter activity was measured and normalized to the internal control β‐galactosidase activity. **(F)** ChIP assay was used to detect the binding of FadR and *adi* promoter regions. C*Δ*fadR-flag** was grown to the logarithmic stage, and DNA fragments were sonicated after washing with PBS. The DNA fragments that interacted with FadR were precipitated with anti-Flag antibodies, and healthy mouse IgG was used as a negative control. The purified DNA fragments were used as PCR templates to amplify the target region of the *adi* promoter. The PCR product of the 16S rRNA gene was used as a negative control. **(G)** The expression of ADI in WT SS2, *Δ*fadR**, FadR-T230A, and FadR-T230E strains were detected by Western blots (left). The band intensity relative to that of WT SS2 group was analyzed (right). The data represent three independent experiments, and are presented as the means ± standard deviations. Statistical analysis was conducted by using One-way ANOVA **(D**, **E**, and **G)**. ^*^, **P* *< 0.05; ^**^, *P* < 0.01; ^***^, *P* < 0.001; ^****^, *P* < 0.0001.

The above studies indicate that FadR and its phosphorylation regulate the acid resistance and virulence of SS2. However, as a transcription factor, it is bound to indirectly exert its regulatory function by regulating downstream target genes. Therefore, to identify the genes regulated by FadR, we used RNA-seq technology to measure the gene expression profiles of *Δ*fadR** and WT SS2 strains. The RT‒qPCR results revealed that when WT SS2 strain was cultured to an OD_600_ of 0.8, the transcription level of *fadR* was the highest (S6 Fig). The total bacterial RNA of WT SS2 and *Δ*fadR** strains during this growth period was extracted and subjected to RNA-seq analysis. The raw data for comparative transcriptomics has been submitted to the SRA database (The BioProject accession number: PRJNA1283375). All comparative transcriptomic results are listed in the S2 Table. Genes with transcription level differences ≥ 2.0 and *P* values ≤ 0.05 were considered to have significant expression differences. Compared with WT SS2 strain, *Δ*fadR** strain contained 88 up-regulated genes and 51 down-regulated genes (Fig 4A). The up-regulated genes were related mainly to carbohydrate metabolism, including hydrolytic enzymes, synthetases, transporter protein permeases, and dehydrogenases (S3 Table). However, other genes involved in transcription, transport, and kinase and deaminase activity were down-regulated in *Δ*fadR** strain (S3 Table). Through KEGG pathway enrichment analysis, it was found that most of these genes were related to glucose metabolism (Fig 4B).

Among all the differentially expressed genes, the gene *adi*, which breaks down arginine, attracted our attention, as it is involved in the acid resistance and virulence of *Streptococcus suis* [30,31]. We first conducted *in vitro* validation experiments using the FadR protein at different concentrations (ranging from 0 ng/μL to 40 ng/μL) and 80 ng of ~300 bp promoter DNA fragments for electrophoretic mobility shift assays (EMSA). The results indicated that the FadR protein could bind to the promoter of *adi*, and the higher the protein concentration was, the more DNA was bound (Fig 4C). To examine whether our *in vitro* observations reflect *in vivo* phenomena, the Flag-tag was fused to the C-terminus of FadR to construct the C*Δ*fadR**-*flag* strain, and the expression of FadR-Flag was detected by Western blotting (S7A Fig). The results of the mouse macrophage phagocytosis experiments revealed that the presence of the Flag-tag did not affect the biological function of SS2 (S7B Fig). In addition, the ChIP results also revealed that FadR can directly bind to the *adi* promoter region *in vivo* (Fig 4F). These findings suggested that FadR may play a role in acid resistance by regulating the transcription of *adi*.

### Phosphorylation of FadR promotes *adi* transcription

To further investigate the effect of phosphorylation on FadR regulation, we used an EMSA to detect the ability of the phosphomimetic protein FadR-T230E to bind to the *adi* promoter. The results showed that compared to the FadR protein, the binding ability of the FadR-T230E protein to the *adi* promoter was significantly enhanced, while the binding ability of the FadR-T230A protein to the *adi* promoter showed no significant difference (Fig 4C). In addition, our study shows that the transcription level of *adi* decreased significantly when SS2 against acid stress, which is similar to the previous report (S8 Fig) [30]. Compared with that of WT SS2 strain, the RT‒qPCR results revealed that the *adi* transcription level of *Δ*fadR** and FadR-T230A strains were significantly decreased, while the *adi* transcription level of FadR-T230E strain was up-regulated approximately 1.5-fold (Fig 4D). Compared with that of WT SS2 strain, Western blot results revealed that ADI protein expression level of *Δ*fadR** and FadR-T230A strains were significantly decreased, while ADI protein expression level of FadR-T230E strain was significantly increased (Fig 4G). To analyze the activity of the *adi* promoter *in vivo*, the pTCV-*lacZ* plasmid was electroporated into WT SS2 and *fadR* variants. As expected, compared with that of WT SS2 strain, the promoter activity of *adi* were significantly decreased in both *Δ*fadR** and FadR-T230A strains, while the promoter activity of *adi* was significantly increased in FadR-T230E strain (Fig 4E). These discoveries indicated that FadR exert positive regulatory effects for *adi*, and phosphorylation further enhances its binding ability to *adi* promoter and also increases the transcription level of *adi*.

### The increased virulence of FadR-T230E strain is due to transcriptional activation of *adi*

**Fig 5:**
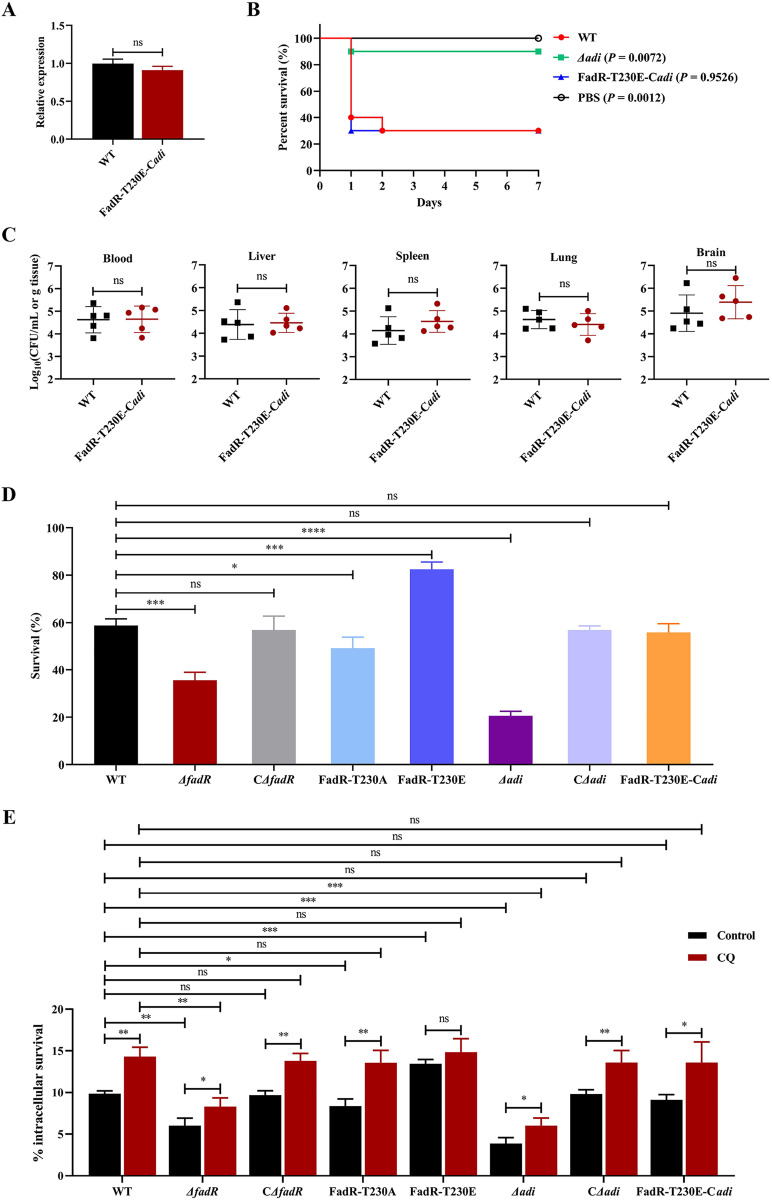
The increased virulence and acid resistance capacity resulting from the phosphorylation of FadR are associated with the promotion of *adi* transcription. **(A)** The *adi* transcript levels in WT SS2 and FadR-T230E-C*adi* strains were determined by RT‒qPCR. **(B)** BALB/c mice were challenged by intraperitoneal injection of WT SS2, *Δ*adi**, and FadR-T230E-C*adi* strains at a dose of 2 × 10^8^ CFUs/mouse (n = 10 mice/group), and the survival time was continuously monitored. The control group is PBS. **(C)** Bacterial loads in the blood, liver, spleen, lung, and brain of BALB/c mice were challenged with 2 × 10^7^ CFUs of WT SS2 and FadR-T230E-C*adi* strains by intraperitoneal injection at 24 hpi (n = 5 mice/group). **(D)** WT SS2, *Δ*fadR**, C*Δ*fadR**, FadR-T230A, FadR-T230E, *Δ*adi**, C*Δ*adi**, and FadR-T230E-C*adi* strains were treated with PBS at pH 5.0 for 1 h, and the number of viable bacteria was subsequently determined by CFU plate counts. The bacterial survival rate was expressed as the ratio of the number of viable bacteria at 1 h to that at 0 h. **(E)** RAW264.7 cells were infected at an MOI of 10:1 with WT SS2, *Δ*fadR**, C*Δ*fadR**, FadR-T230A, FadR-T230E, *Δ*adi**, C*Δ*adi**, and FadR-T230E-C*adi* strains for 1 h. After 1 h of antibiotic sterilization, the amount of internalized bacteria was regarded as 100% to calculate percent survival. After 3 h of antibiotic sterilization, sterile water was used to lyse the cells to release bacteria. Then bacteria were serial-diluted in PBS buffer and spread onto THY plates, incubated at 37°C for 16 h. Black bars correspond to the control group, and red bars correspond to the CQ group. The data were representative of three independent experiments and are presented as the means ± standard deviations. Statistical analysis was performed by using an unpaired *t*-test **(A and C)**, Long-rank test **(B)**, One-way ANOVA **(D)**, and Two-way ANOVA **(E)**. ^ns^, *P >* 0.05; ^*^, **P* *< 0.05; ^**^, **P* *< 0.01; ^***^, **P* *< 0.001; ^****^, **P* *< 0.0001.

At present, it has been shown that *adi* was an important virulence factor of bacteria [32–34]. Therefore, we suspected that the increased virulence of the FadR-T230E strain was related to the increased transcription of *adi*. The promoter of *impdh* [35] was fused to the *adi* gene sequence, after which the pSET2-imp-*adi* plasmid was constructed to avoid the regulatory effect of FadR on *adi* [24]. The complemented plasmid (pSET2-imp-*adi*) was electroporated into FadR-T230E competent cells lacking *adi* gene to obtain the FadR-T230E-C*adi* strain. In addition, we tested the growth curves of the strains and confirmed that carrying the plasmid did not affect its growth rates (S9 Fig). The RT‒qPCR results revealed that the transcription level of *adi* in the FadR-T230E-C*adi* strain was not significantly different from that in WT SS2 strain (Fig 5A). In addition, animal experimental model revealed that, compared with that of the mice injected with WT SS2 strain (30%), the survival rate of the mice injected with *Δ*adi** (90%) strain was significantly increased, while the survival rate of the mice injected with the FadR-T230E-C*adi* (30%) strain showed no significant difference (Fig 5B). Meanwhile, the CFUs of the blood and various organs in the mice infected with FadR-T230E-C*adi* strain in mice decreased to WT SS2 strain level (Fig 5C). Overall, these above results indicated that the increased virulence of SS2 caused by STK/FadR phosphorylation pathway is due to the increased transcription of *adi*.

### FadR phosphorylation affects acid resistance capacity and survival in host macrophages

The *adi* is involved in bacterial acid resistance [14,30,36], whether FadR phosphorylation affects the acid resistance of SS2 by regulating *adi* needs further exploration. Here, to demonstrate the correlation between FadR phosphorylation and the acid resistance of SS2, we compared the survival ability of WT SS2, *fadR* variants, and *adi* variants under acid stress conditions. The results showed that after 1 h of stimulation in a PBS environment at pH 5.0, compared with that of WT SS2 strain, the survival rate of *Δ*fadR**, FadR-T230A, and *Δ*adi** strains were significantly decreased, the survival rate of the FadR-T230E strain was significantly increased, while the survival rates of C*Δ*fadR**, C*Δ*adi**, and FadR-T230E-C*adi* strains showed no significant difference (Fig 5D). In addition to its acid resistance, bacteria can also produce the acid tolerance response (ATR) [16,37]. To investigate whether the STK/FadR signaling pathway affects SS2’s ATR, we first measured the sub-lethal pH and lethal pH in WT strain (S10A Fig). Subsequently, we compared ATR levels among WT SS2, *Δ*stk**, *Δ*fadR**, FadR-T230A, and FadR-T230E strains. The results showed that after sub-lethal pH treatment, the survival rates of WT SS2 significantly increased, while those of *Δ*stk**, *Δ*fadR**, FadR-T230A, and FadR-T230E strains remained unchanged (S10B Fig). Therefore, the above results indicate that STK enhances the acid resistance and ATR of SS2 by phosphorylation of FadR, and the effects of FadR phosphorylation on acid resistance of SS2 were closely related to the increase in *adi* transcription.

Many pathogens have the ability to survive for a period of time in a membrane-bound acidic chamber within macrophages [38]. Therefore, we further investigated whether FadR phosphorylation enhances the intracellular survival ability of SS2 in RAW264.7 macrophages. After lysosome acidification, an acidic pH environment (typically ranging between pH 4.5 and pH 5.0) is formed, which subsequently kills and degrades pathogens [39]. Intracellular survival was measured in the absence or presence of Chloroquine (CQ), an effective inhibitor of lysosomal acidification, to investigate the effect of the macrophage lysosomal acidification on WT SS2 and mutant strains. In the absence of CQ, compared with that of WT SS2 strain, the intracellular survival rate of *Δ*fadR**, FadR-T230A, and *Δ*adi** strains were significantly decreased, the intracellular survival rate of FadR-T230E strain was significantly increased, while the intracellular survival rates of C*Δ*fadR**, C*Δ*adi**, and FadR-T230E-C*adi* strains showed no significant difference (Fig 5E). In the presence of CQ, the intracellular survival rates of all strains increased, with no significant difference observed between WT SS2, C*Δ*fadR**, FadR-T230A, FadR-T230E, C*Δ*adi**, and FadR-T230E-C*adi* strains, while the intracellular survival rates of *Δ*fadR** and *Δ*adi** strains did not fully recover to WT SS2 level (Fig 5E). The treatment with CQ eliminated the survival defect of SS2 within macrophages, revealing that the bactericidal effect of macrophage lysosomal acidification was effectively neutralized or weakened by the FadR phosphorylation pathway. In summary, the above results indicated that the STK/FadR pathway not only enhances the acid resistance of the bacteria but also significantly improves their ability to survive in RAW264.7 macrophages.

### The mechanism of SS2 utilizing the STK/FadR axis to resist acid and promote macrophage survival

**Fig 6:**
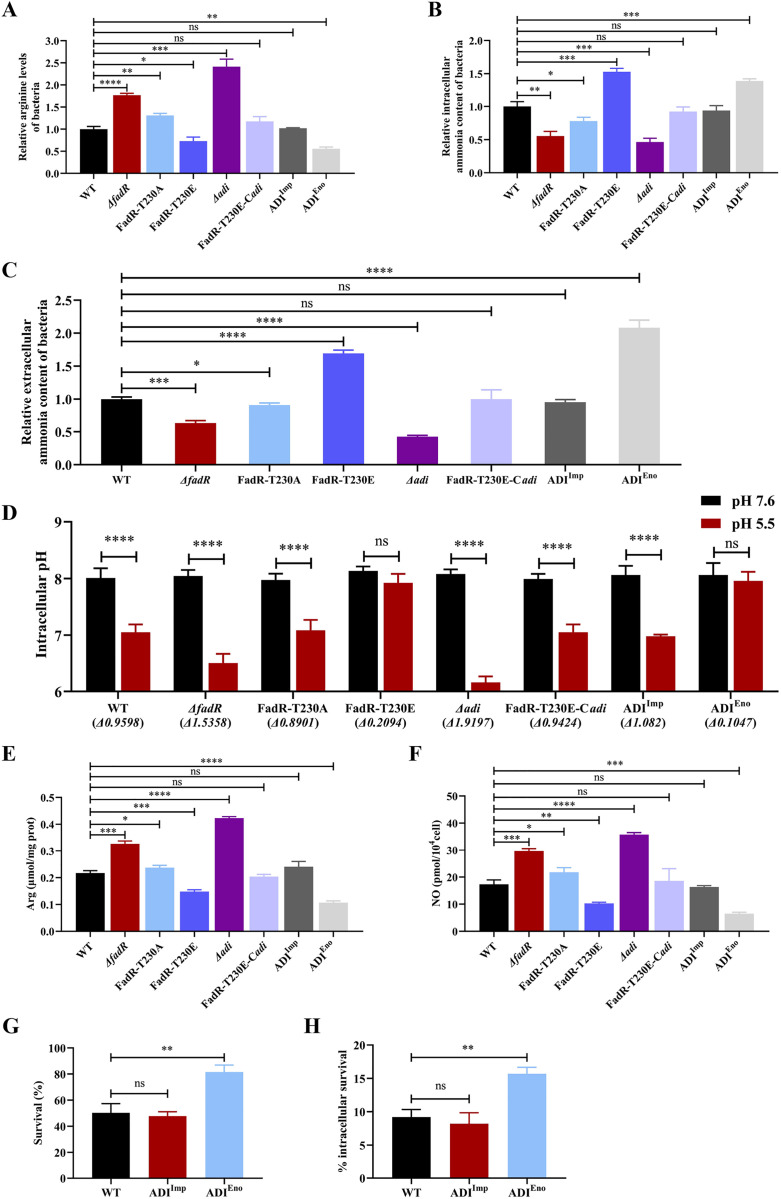
Molecular mechanism of acid resistance in SS2. **(A)** WT SS2, *Δ*fadR**, FadR-T230A, FadR-T230E, *Δ*adi**, FadR-T230E-C*adi*, ADI^Imp^, and ADI^Eno^ strains were placed in pH 5.5 PBS and incubated for 1 h before the levels of intracellular arginine were measured. **(B)** Intracellular ammonia contents of WT SS2, *Δ*fadR**, FadR-T230A, FadR-T230E, *Δ*adi**, FadR-T230E-C*adi*, ADI^Imp^, and ADI^Eno^ strains were determined according to the method used in **(A)**. **(C)** The ammonia released by WT SS2, *Δ*fadR**, FadR-T230A, FadR-T230E, *Δ*adi**, FadR-T230E-C*adi*, ADI^Imp^, and ADI^Eno^ strains were measured using the method described in **(A)**. **(D)** WT SS2, *Δ*fadR**, FadR-T230A, FadR-T230E, *Δ*adi**, FadR-T230E-C*adi*, ADI^Imp^, and ADI^Eno^ strains were placed in pH 7.6 PBS to measure the intracellular pH, then quickly transferred to pH 5.5 PBS and incubated for 1 h before the intracellular pH was measured again. **(E)** WT SS2, *Δ*fadR**, FadR-T230A, FadR-T230E, *Δ*adi**, FadR-T230E-C*adi*, ADI^Imp^, and ADI^Eno^ strains were added into the RAW264.7 cell holes at an MOI of 100:1 incubating for 1 h. The hole without strain was used as the control. The culture medium in these wells was replaced with DMEM containing gentamicin (100 µg/mL) and penicillin (10 µg/mL). After 3 h of antibiotic sterilization, the arginine content produced by RAW264.7 was determined using an arginine assay kit. **(F)** WT SS2, *Δ*fadR**, FadR-T230A, FadR-T230E, *Δ*adi**, FadR-T230E-C*adi*, ADI^Imp^, and ADI^Eno^ strains were added into the RAW264.7 cell holes at an MOI of 100:1 incubating for 1 h. The culture medium in these wells was replaced with DMEM containing gentamicin (100 µg/mL) and penicillin (10 µg/mL). The NO content produced by RAW264.7 cells was determined using a micro NO content assay kit after 24 h. **(G)** WT SS2, ADI^Imp^, and ADI^Eno^ strains were treated with PBS at pH 5.0 for 1 h, and the number of viable bacteria was subsequently determined by CFU plate counts. The bacterial survival rate was expressed as the ratio of the number of viable bacteria at 1 h to that at 0 h. **(H)** RAW264.7 cells were infected at an MOI of 10:1 with WT SS2, ADI^Imp^, and ADI^Eno^ for 1 h. After 1 h of antibiotic sterilization, the internalized bacterial count was regarded as 100% to calculate percent survival. After 3 h of antibiotic sterilization, sterile water was used to lyse the cells to release bacteria. Then bacteria were serial-diluted in PBS buffer and spread onto THY plates, incubated at 37°C for 16 h. The data shown represent three independent experiments and are presented as the means ± standard deviations. Statistical analysis was performed by One-way ANOVA **(A**-**C** and **E**-**H)** and Two-way ANOVA followed by Bonferroni’s multiple comparisons test **(D)**. ^ns^, *P* > 0.05; ^*^, *P* < 0.05; ^**^, *P* < 0.01; ^***^, **P* *< 0.001; ^****^, **P* *< 0.0001.

The arginine deaminase system can decompose arginine, ultimately producing substances such as ornithine and ammonia, thereby increasing the acid resistance of bacteria [13]. Meanwhile, we used the promoters of *impdh* (weak promoter) [35] and *enolase* (strong promoter) [40] to control the expression of ADI in SS2, and then constructed knockdown strain ADI^Imp^ (replace the promoter of *adi* with weak promoter *impdh*) and overexpression strain ADI^Eno^ (replace the promoter of *adi* with strong promoter *enolase*) of ADI to more accurately investigate the effect of ADI content on SS2 virulence. In addition, we tested the growth curves of the strains and confirmed that carrying the plasmid did not affect their growth rates (S9 Fig). We measured the arginine content in bacteria and found that compared with WT SS2 strain, the arginine content in *Δ*fadR**, FadR-T230A, and *Δ*adi** strains were significantly increased, the arginine content in FadR-T230E and ADI^Eno^ strains were significantly decreased, while the arginine content in FadR-T230E-C*adi* and ADI^Imp^ strains showed no significant difference (Fig 6A). *In vitro* enzymatic reaction assays were performed, and the results showed that recombinant arginine deaminase could decompose arginine, which further confirmed our hypothesis (S11 Fig) [30]. To further determine the ammonia production of bacteria, we first established a standard curve with an ammonia content detection kit, and the results revealed an almost linear relationship between the ammonia concentration and fluorescence intensity (S12A Fig). We measured the ammonia content inside and outside the bacteria. The results revealed that the ammonia contents of FadR-T230E and ADI^Eno^ significantly increased compared with those of WT SS2, the ammonia contents of *Δ*fadR**, FadR-T230A, and *Δ*adi** strains significantly decreased compared with those of WT SS2, whereas the ammonia levels in FadR-T230E-C*adi* and ADI^Imp^ were the same as those in WT SS2 (Fig 6B and 6C). Altogether, these results indicated that the increase of ADI accelerates the conversion of arginine to ammonia, which in turn improves the survival ability of SS2 in acidic environments.

To resist acidic environments and improve their intracellular survival ability, most bacteria are able to grow in the external pH range of 5.5–9.0 and maintain the cytoplasmic pH within a suitable range of 7.4–7.8 [41]. On the basis that the phosphorylation of FadR enhances the acid resistance of WT SS2, we speculated that phosphorylation of FadR may affect the intracellular pH of WT SS2. To eliminate the influence of acidic stimuli on bacterial death, a weakly acidic environment (pH 5.5) was chosen, and the survival of *Δ*adi** was significantly lower than that of WT SS2 (S13A Fig). The survival ability of *Δ*adi** in PBS at pH 5.5 at different time points was tested, and the results revealed that *Δ*adi** presented little change up to 30 minutes but significantly decreased at 40 minutes after treatment (S13B Fig). We first used extracellular buffer to balance the pH inside the SS2 cells to establish a standard curve, and the results revealed an almost linear relationship between the intracellular fluorescence and pH (S12B Fig). Indeed, when bacteria were quickly switched from pH 7.6 to pH 5.5 and incubated at 37°C for 1 h, the decrease in the intracellular pH of WT SS2 was ~ 1 unit, which was more than 0.5 units greater than that experienced by FadR-T230E and ADI^Eno^ (Fig 6D). Additionally, the intracellular pH of *Δ*fadR**, FadR-T230A, *Δ*adi**, FadR-T230E-C*adi*, and ADI^Imp^ strains also decreased significantly (Fig 6D). These results indicate that *adi* can assist SS2 in maintaining stable intracellular pH.

Many pathogens have the ability to resiliently survive in the acidic microenvironment created by macrophages [38]. Such as, bacteria can utilize intracellular arginine to resist acid-mediated killing [30]. To further mimic *in vivo* infections, all strains were co-incubated with RAW264.7 macrophages for 3 h. Compared to WT SS2 strain, the intracellular arginine levels were significantly increased in cells infected with *Δ*fadR**, FadR-T230A, and *Δ*adi** strains, the intracellular arginine levels were significantly decreased in cells infected with FadR-T230E and ADI^Eno^ strains, while no significant differences in arginine content were observed in cells infected with FadR-T230E-C*adi* and ADI^Imp^ strains, suggesting that the ADI pathway can consume intracellular arginine (Fig 6E). Given that arginine in macrophages is also an important source of nitric oxide (NO), we detected the concentration of NO in the cells. The results showed that the production of NO were significantly increased in cells infected with *Δ*fadR**, FadR-T230A, and *Δ*adi** strains compared to WT SS2 strain, the production of NO were significantly decreased in cells infected with FadR-T230E and ADI^Eno^ strains, while no significant differences for NO production were observed in cells infected with FadR-T230E-C*adi* and ADI^Imp^ strains (Fig 6F). Importantly, ADI^Eno^ strain had a similar phenotype to FadR-T230E strain. Therefore, these data further show that the enhanced virulence of SS2 resulting from FadR phosphorylation is due to increased transcription of *adi*. In summary, our study demonstrated that FadR phosphorylation upregulates the transcription level of *adi*, thereby enhancing the ability of SS2 to acquire arginine from host cells, ultimately leading to increased ammonia production that enhances acid tolerance and intracellular survival capacity.

Meanwhile, we tested the acid tolerance of knockdown strain ADI^Imp^ and overexpression strain ADI^Eno^ of ADI. The results showed that compared to WT SS2 strain, the survival rate of ADI^Eno^ strain was significantly increased, while the survival rate of ADI^Imp^ strain was similar to that of WT SS2 strain (Fig 6G). We further examined the intracellular survival capacity of ADI^Imp^ and ADI^Eno^ strains. The results indicated that, compared to WT SS2 strain, the survival rate of ADI^Eno^ strain was significantly improved, whereas the survival rate of ADI^Imp^ strain was similar to that of WT SS2 strain (Fig 6H). The research results indicated that the enhanced acid tolerance and intracellular survival capacity of FadR-T230E strain are attributed to the increased transcription of *adi*.

We further investigated the link between STK and *adi*, as STK has been implicated in the acid resistance process of *Streptococcus suis* [35]*.* Our study showed that the acid resistance of *Δ*stk** strain significantly decreases, while the acid resistance of C*Δ*stk** strain has basically recovered to WT SS2 strain level (S14A Fig). To further demonstrate the regulatory effect of STK on *adi* in acidic conditions, WT SS2, *Δ*stk**, and C*Δ*stk** strain were incubated at 37°C for 1 h in TYH at pH 5.5, and then RNA-seq technology was used to detect the transcription levels of *adi* in WT SS2 strain, *Δ*stk**, and C*Δ*stk** strain. The RT‒qPCR results showed that, in comparison to WT SS2 strain, the transcription level of *adi* is significantly decreased in *Δ*stk** strain, while the transcription level of *adi* in C*Δ*stk** strain was restored (S14B Fig). In addition, Western blotting was used to assess the expression level of ADI protein. The results indicated that, in comparison to WT SS2 strain, the expression level of ADI protein is significantly reduced in *Δ*stk** strain, while the expression of ADI protein in C*Δ*stk** strain was decreased (S14C Fig). These results suggested that STK affect the transcription and expression levels of *adi*, thereby participating in the SS2 acid resistance process. Altogether, the STK indirectly enhances the transcript level of *adi* by phosphorylating FadR, which in turn enhances bacterial acid tolerance and virulence.

## Discussion

**Fig 7:**
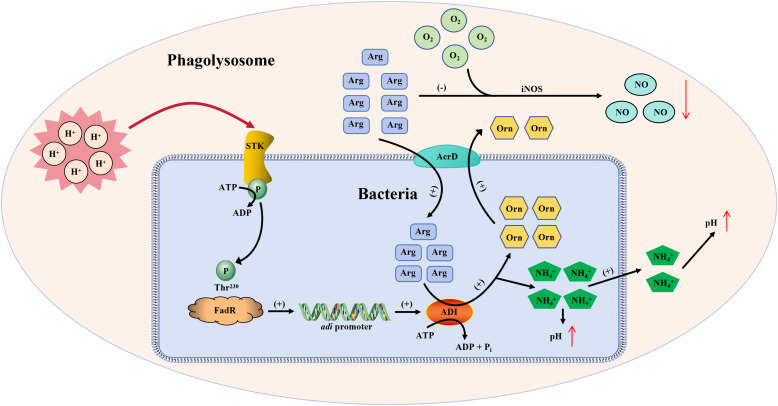
A model schematic depicting for STK/FadR pathway that the impacts on acid stress and intracellular survival mechanisms in *S. suis* exposed to an acidic environment.

The survival of bacteria in the face of host macrophage phagocytosis and killing largely depends on their acid resistance ability [5,42]. To date, how bacteria perceive extracellular signals to regulate their acid tolerance remains elusive. Here, our study indicate that bacteria have evolved intricate acid resistance mechanisms, establishing a linkage between the STK/STP signal transduction system and the arginine deaminase acid-base regulatory system. In our model (Fig 7), FadR, as a cornerstone, not only receiving signals from STK in response to extracellular stimuli, but also converting these signals into specific physiological functions and regulating the transcription level of *adi*. The increase in ADI protein levels in bacteria accelerates the conversion of arginine to ammonia, enhances its acid resistance, and thus maintains pH stability in bacteria. These findings reveal an important acid resistance mechanism: bacteria rapidly respond to acidic environments through the STK-FadR axis and regulate ADI levels to stabilize pH in bacteria, thereby enhancing bacterial acid resistance and virulence.

In our previous studies, some phosphorylation substrate proteins of SS2 STK have been identified, including GntR, GlmM, and CcpS [24,25,43]. In this study, compared with WT strain, FadR phosphorylation disappeared in *Δ*stk** strain, while FadR phosphorylation recovered to WT strain level in C*Δ*stk**, indicating that SS2 STK can affect FadR phosphorylation. In addition, compared with WT strain, the phosphorylation level of FadR in FadR-T230A strain was significantly reduced, but complete absence of FadR phosphorylation was not observed. This could be due to non-specific binding appearing in antibodies targeting phosphorylation sites of prokaryotic proteins [16,24]. Importantly, we have confirmed through *in vitro* phosphorylation assays that FadR is indeed a direct substrate of STK in SS2, with the phosphorylation site being Thr-230, which is consistent with the results of the phosphorylation mass spectrometry analysis (S1 Fig). Of course, it cannot be completely ruled out that FadR may have other phosphorylation sites.

The STK was involved in the acid resistance process of *Streptococcus suis*, although the specific mechanism remains unclear [35]. FadR, as a new direct substrate of the STK system, exhibits different phosphorylation patterns in SS2, further confirming the important role of STK in bacterial biological signaling and regulation. Interestingly, the phosphorylation level of FadR significantly increased under acid stress or oxidative stress (Fig 2), indicating that the STK‒FadR axis may be an important signaling pathway that helps bacteria resist adverse environments. It is noteworthy that our previous research has also shown that phosphorylation of the STK‒CcpS axis is active when bacteria confront various stressors [25]. However, the specific molecular mechanism by which bacterial STK directly or indirectly senses acid stress signals and transmits them to substrate proteins remains to be further investigated.

Previous studies have shown that STK-mediated GntR phosphorylation reduces the virulence and pathogenicity of SS2 in mice [24]. Our research revealed that, compared with that of the mice injected with WT SS2 strain (30%), the survival rate of the mice injected with *Δ*fadR** (80%) and FadR-T230A (60%) strains were significantly increased, while the survival rate of the mice injected with FadR-T230E (0%) strain was significantly decreased (Fig 3A). In addition, compared with WT SS2 strain, the CFUs of the blood and various organs in the mice infected with *Δ*fadR** and FadR-T230A strains were significantly decreased, while the CFUs of the blood and various organs in the mice infected with FadR-T230E strain were significantly increased (Fig 3B–3F). Meanwhile, compared with *Δ*fadR** strain, the CFUs of the blood and various organs in the mice infected with FadR-T230A strains were significantly increased. The above results indicate that FadR is essential for the virulence of SS2, and that FadR phosphorylation significantly boosts the virulence and pathogenicity of SS2. Moreover, compared to *Δ*fadR** strain, FadR-T230A strain showed higher lethality in mice and increased bacterial loads in the blood and organs of mice. Our studies show that FadR positively regulates *adi* and FadR can still bind to *adi* promoter in non-phosphorylated state, but the binding ability of non-phosphorylated FadR to the *adi* promoter is weak, while phosphorylated FadR to the *adi* promoter is greatly enhanced, thus significantly enhancing the transcription of *adi* and the virulence of SS2. This could be attributed to the fact that, despite phospho-ablative FadR-T230A mutant strain cannot further enhance *adi* transcription via phosphorylation, it can still regulate *adi* transcription at a lower level. Therefore, it is easier to understand that FadR-T230A still has a certain effect on SS2 virulence compared to *Δ*fadR** strain, because *Δ*fadR** strain completely loses its regulatory function over *adi*.

Many pathogens have the ability to survive for a period of time in a membrane-bound acidic chamber within macrophages [38]. After lysosomal acidification, an acidic environment is formed to kill pathogens [39]. Our study indicates that FadR and its phosphorylation can enhance the intracellular survival of SS2 in RAW264.7 macrophages. In the presence of CQ, the intracellular survival ability of all strains was significantly increased, with no significant difference observed between WT SS2, C*Δ*fadR**, FadR-T230A, FadR-T230E, C*Δ*adi**, and FadR-T230E-C*adi* strains, while the intracellular survival rates of *Δ*fadR** and *Δ*adi** strains did not fully recover to WT SS2 level (Fig 5E). Macrophages have evolved a variety of defense strategies to combat and kill bacteria [39]. Comparative transcriptomics results showed that compared with WT SS2 strain, there were 139 genes with significant differences in *Δ*fadR** strain, which, in addition to acid resistance, are also involved in bacterial metabolism and other virulence regulation, thereby affecting the intracellular survival ability of SS2. The *adi* is involved in the acid resistance and virulence of bacteria [14,30,36]. The arginine deiminase system can metabolize arginine into ornithine, ammonia, and carbon dioxide [31]. One of the metabolites, ornithine, can help RocR (the transcription activator of the roc gene) to show ATP enzyme activity, which induces the expression of the *roc* gene [44]. Additionally, studies have shown that Ornithine affects the formation of bacterial biofilms [45,46]. Therefore, *Δ*adi** strain could not convert arginine into ammonia and ornithine, which not only resulted in decreased acid resistance of SS2, but also the decrease of ornithine might similarly affect the formation of bacterial biofilms. Therefore, the different virulence of *Δ*adi** and WT SS2 strains in the presence of CQ may be related to the intersection of *adi* metabolic pathway with other metabolic circuits, indicating that the function of *adi* is complex.

FadR belongs to the GntR transcription factor subfamily, showing a typical receiver domain and a DNA-binding domain [47–50]. Here, we identify FadR as a potential phosphorylation substrate of STK, suggesting that the GntR family can receive phosphorylation signal regulatory from STK, thereby offering new insights into the functional regulation of this transcription factor family. Interestingly, the phosphorylation modification of FadR occurs at the T230 residue within its C-terminal effector binding domain. We speculate that this localization suggests a mechanism whereby phosphorylation of these residues activates the effector region of the transcription factor, leading to conformational changes in the DNA-binding domain and thereby enhancing its interaction with the promoter sequences of downstream target genes [19]. However, the specific molecular mechanism by which FadR’s carboxyl terminal phosphorylation alters its binding ability to DNA still needs further investigation.

XRE family transcriptional regulator XtrSs can inhibit ADI system by regulating ArgR, thereby reducing the acid resistance and virulence of *Streptococcus suis* [30]. Interestingly, our research indicated that FadR positively regulates the expression of ADI, enhancing the acid tolerance and virulence of SS2. This discovery implied that the *adi* gene is subject to multiple regulations, and simultaneously indicated the existence of a complex regulatory network in the process of SS2 responding to acid stress. In addition, we found that the transcription level of *adi* decreased significantly when SS2 against acid stress, which is similar with the report as above. Notably, in neutral environments, FadR phosphorylation levels in WT SS2 are lower, resulting in weak *adi* transcriptional regulation. However, in acidic stress, FadR phosphorylation in SS2 significantly enhanced, thereby enhancing *adi* transcription (Fig 2). Importantly, our study reveals that compared with phospho-ablative strain FadR-T230A, the survival rate of the phosphomimetic strain FadR-T230E was significantly increased in acid stress conditions (Fig 5D). Altogether, these findings reveal that FadR phosphorylation is a very important pathway to enhance the transcription and protein levels of *adi*, which assists SS2 maintaining survival in acidic stress conditions.

The ADI system has been described in many streptococcal species, such as *Streptococcus pneumoniae* and *Streptococcus pyogenes*, encompassing a wide range of biological functions [34]. The ADI system converts arginine into ornithine through a two-step reaction, generating carbamoyl phosphate and ammonia, and the ammonia combines with hydrogen ions to produce ammonium ions, thereby increasing the pH value within the bacterial cells [42]. However, it is still not fully understood how bacteria manipulate the ADI system to respond to host immunity or adverse environments. We found that FadR regulates the transcription of *adi*, and phosphorylated FadR further enhances *adi* transcription. The increase in ADI expression level accelerates the conversion of arginine to ammonia, ultimately stabilizing intracellular pH (Fig 6). The ADI system can utilize arginine to enhance the acid resistance of *Streptococcus suis* [30], while the phosphorylation regulation of STK-FadR enables *Streptococcus suis* to more effectively perceive the extracellular acidic environment and manipulate the ADI system to resist unfavorable host environments, enhancing its survival ability. In summary, SS2 can sense the extracellular acidic environment through the STK/STP system, and then phosphorylates FadR, and regulates its transcriptional activity and ADI levels, which is beneficial for bacteria to adapt flexibly and quickly to acidic environments and host niches.

Most pathogens typically adopt two different strategies to survive in macrophage acidic phagosomes: they actively alter the pH of the phagosome or resist the consequences of phagosome acidification [5]. We have demonstrated that SS2 can utilize STK to sense changes in acidic environments, activate FadR activity through phosphorylation, and increase ADI expression levels, accelerating the conversion of arginine to ammonia. The increase in ammonia content helps SS2 resist phagocytic acidification. Nitric oxide (NO), as an important immune regulatory factor, can enhance the activity of macrophages, thereby more effectively engulfing and clearing pathogens and their products [51]. Arginine metabolism also contributes to M1 polarization of macrophages, thereby resisting pathogen infection [52]. Studies have shown that the production of L-arginine-dependent reactive nitrogen species is a major mechanism for killing and inhibiting *Mycobacterium tuberculosis* in mouse macrophages [53,54]. Similarly, the ADI system of *Streptococcus suis* can compete with cellular iNOS for arginine to counteract the production of NO and resist innate immune killing [30]. Our research results indicate that SS2 can flexibly manipulate the ADI system through the STK/FadR axis in the face of acidic environments or macrophage phagocytic killing, accelerating the conversion of bacterial arginine to ammonia. However, it consumes arginine in mouse macrophages, leading to a decrease in intracellular NO and a decrease in macrophage phagocytic killing ability, thereby enhancing bacterial intracellular survival ability. These results further indicate that phosphorylation regulation is crucial for the intracellular survival of SS2.

The published study has shown that in normal THY medium, the transcription level of *adi* in the *Δ*stk** strain is decreased compared to WT SS2 strain, indicating that *adi* transcription is related to STK activity [35]. In our study, we have revealed that the STK-FadR-ADI regulation pathway, in which STK directly phosphorylates FadR, and FadR positively regulates of transcription and expression levels of *adi*. Further studies reveals that the binding ability of non-phosphorylated FadR to the *adi* promoter is weak, while phosphorylated FadR to the *adi* promoter is greatly enhanced, thus significantly enhancing the transcription level of *adi* (Fig 4). Our research reveals that in acidic THY medium, the transcriptional level of *adi* in *Δ*stk** strain is markedly lower than that in WT SS2 strain, while C*Δ*stk** strain is restored to WT SS2 level (S14 Fig). In neutral environments, FadR phosphorylation levels in WT SS2 are lower, resulting in weak *adi* transcriptional regulation. However, in acidic stress, FadR phosphorylation in SS2 significantly enhanced, thereby enhancing *adi* transcription. These findings show that STK-FadR-ADI regulation is acid-dependent. Therefore, it is easy to understand that the strain in absence of *stk* gene can’t sense acidic signal, leading to FadR being in a non-phosphorylated state and ultimately resulting in *adi* low transcription levels. These results indicate that STK indirectly regulates the transcription and expression of *adi* by phosphorylating FadR, thereby participating in the acid resistance process of SS2.

In conclusion, our research demonstrates that FadR serves as a cornerstone, receiving signals from upstream STK and coordinating bacterial acid resistance. This biological process can help bacteria perceive changes in acidic environments and effectively regulate the internal pH stability of bacteria, thereby enhancing their resistance to killing by host macrophages and ultimately augmenting the virulence of pathogens. The study provides new insights into the mechanism by which SS2 perceives the extracellular environment and regulates its acid tolerance, thereby resisting the killing of host immune cells.

## Materials and methods

### Ethics statement

All animal experiments were approved by the Laboratory Animal Welfare and Ethics Committee of Nanjing Agricultural University, China (approval number NJAU.No20210510065 and NJAU.No20211005144). The Chinese National Laboratory Animal Guideline for Ethical Review of Animal Welfare adhered to animal care and protocol.

### Bacterial strains, plasmids, cell lines, and growth conditions

The bacterial strains and plasmids used in this study are listed in the S4 Table. The SS2 strain ZY05719 was isolated from dead pigs infected with *Streptococcus suis* in Sichuan Province, China. SS2 was grown at 37°C in THY (Todd-Hewitt Broth [THB, Becton, Franklin Lakes, NJ, USA] supplemented with 2% yeast extract [Oxoid Ltd., UK]) or in THA (THY containing 1.5% agar). *E. coli* DH5α was used as the host for the preparation of plasmid DNA and was cultured at 37°C in lysogeny broth (LB, Oxoid Ltd.) or LB containing 1.5% agar. *E. coli* BL21 (DE3) was used for protein prokaryotic expression and was cultured at 37°C in lysogeny broth (LB, Oxoid Ltd.) or LB containing 1.5% agar. Spectinomycin (Spc) was used at 50 μg/mL for *E. coli* and 100 μg/mL for SS2. Gentamicin (100 μg/mL) and penicillin (10 μg/mL) were used to kill the extracellular SS2. RAW264.7 cells were grown in DMEM containing 10% fetal calf serum in an incubator at 37°C and 5% CO_2_.

### Strain construction

In this study, a deletion vector and a replacement vector were constructed by homologous recombination. With the ZY05719 genome as a template, the upstream and downstream homologous arm fragments of FadR were amplified by PCR with the primers *Δ*fadR**-F1/*Δ*fadR**-R1 and *Δ*fadR**-F2/*Δ*fadR**-R2. The two PCR products were purified by gel recovery, and the two purified products were subsequently mixed at a ratio of 1:1 and amplified by fusion PCR with the using primers *Δ*fadR**-F1/*Δ*fadR**-R2. The fusion fragment was subsequently cloned and inserted into the temperature-sensitive shuttle vector pSET4s for SS2 ZY05719 and *E. coli* with the ClonExpress II One Step Cloning Kit (Vazyme Biotech Co., China; item number: C112-01) to construct the recombinant vector pSET4s-*fadR*. In addition, the plasmids pSET4s-*adi* and pSET4s-*stk* were obtained by the same method. With the ZY05719 genome as a template, the *fadR* and its upstream and downstream homologous arm fragments full-length were amplified by PCR with the primers *fadR*-F/*fadR*-R. The template plasmid pSET4s-(homologous arm)-*fadR* for in situ point mutation was obtained by the same method. With the plasmid pSET4s-(homologous arm)-*fadR* as a template, PCR amplification was performed with the primers FadR-T230A-F/R and FadR-T230E-F/R, respectively. The template circular plasmid in the PCR product was digested with DpnI enzyme, after which pSET4s-*fadR*-T230A and pSET4s-*fadR*-T230E constructs were obtained. The pSET4s-*fadR*, pSET4s-*adi*, and pSET4s-*stk* were electroporated into SS2 ZY05719 with the Gene Pulser XCell electroporation system (Bio-Rad, Hercules, CA, USA) (voltage: 2300 V, capacitance: 25 μF, resistance: 200 Ω, cuvette: 1 mm). To obtain *Δ*fadR** and *Δ*adi**, it was necessary to first perform subculturing in a 28°C incubator and then perform PCR identification, which is consistent with previous methods [55,56]. The pSET4s-*fadR*-T230A and pSET4s-*fadR*-T230E plasmids were electroporated into *Δ*fadR** strain, and the in situ mutant strain of *fadR* was obtained by the above method.

The complementary strain of the *Δ*fadR** mutant was constructed as follows. With the SS2 ZY05719 genome as a template, PCR amplification was performed with the primers C*Δ*fadR**-F/C*Δ*fadR**-R to obtain the *fadR* fragment, which was then cloned and inserted into the plasmid pSET2. Subsequently, the recombinant plasmid was electroporated into the *Δ*fadR** strain to obtain the C*Δ*fadR** strain. The C*Δ*fadR**-*flag* and C*Δ*adi** strain was obtained in the same manner. The ADI overexpressing strains were constructed as follows. With the SS2 ZY05719 genome as a template, the *adi* fragment sequence was amplified by PCR and fused to the promoter sequences of *impdh* and *enolase*, respectively. Subsequently, these two fusion fragments were separately cloned into pSET2 plasmid and electroporated into *Δ*adi** to obtain low expression strain ADI^Imp^ and overexpression strain ADI^Eno^ of ADI. In addition, the *impdh*-*adi* was cloned into pSET2 plasmid and electroporated into FadR-T230E-*Δ*adi** to obtain transcriptional recovery strain FadR-T230E-C*Δ*adi** of ADI. All strains and plasmids used are listed in the S4 Table. All primers used are listed in the S5 Table.

### Phosphoproteomics analysis

WT SS2 and *Δ*stk** strain were cultured under shaking at 37°C until logarithmic growth stage (OD_600 _= 0.4 ~ 0.6), 8000 rpm, 4°C, and centrifuged for 10 minutes to collect bacterial cells. Each strain provides three biological replicate samples. The bacterial cell precipitation was washed twice with ice cold PBS, and then stored at low temperature and transported to Hangzhou PTM Bio-Tech company for phosphoproteomics analysis. Identification of phosphorylated proteins and their corresponding phosphorylation sites in SS2 using phosphorylation modified 4D Label free quantitative proteomics, and comparison of whole protein phosphorylation status between two bacterial strains.

### Protein expression, purification, and preparation of polyclonal antibodies

The *stk* gene fragment sequence was amplified by PCR from the ZY05719 genome with the primers nSTK-F/nSTK-R, and subsequently cloned and inserted into the expression vector pGEX4T-1. With the SS2 ZY05719 genome as a template, the gene sequences of *fadR* and *adi* were amplified by PCR using primers B-FadR-F/B-FadR-R and ADI-F/ADI-R, respectively. Subsequently, the amplified sequences were cloned and inserted into the expression vector pET28a, resulting in the construction of plasmids pET28a-*fadR* and pET28a-*adi*. Plasmids pET28a-*gapdh* and pET28a-*groel* were obtained by using the same method. With the plasmid pET28a-*fadR* as a template, PCR amplification was performed with the primers B-FadR-T230A-F/R and B-FadR-T230E-F/R. The template circular plasmid in the PCR product was digested with DpnI enzyme, after which pET28a-FadR-T230A and pET28a-FadR-T230E constructs were obtained. All the plasmids were first transformed into *E. coli* DH5α, after which the positive plasmids were extracted and transformed into *E. coli* BL21 (DE3) to obtain recombinant expression strains. The GST-tagged proteins were purified with GST-tag purification resin (Beyotime, Shanghai, China), and the His-tagged proteins were purified with HisTrap HP (5 mL; GE Healthcare, Piscataway, NJ, USA). BALB/c mice were immunized by subcutaneous injection of 100 μg of the purified FadR protein, ADI protein, GAPDH protein, and GroEL protein emulsified with adjuvants. All immunizations were administered every 2 weeks for a total of 3 times, and serum samples were collected from the mice a week after the third immunization. All strains and plasmids used are listed in the S4 Table. All primers used are listed in the S5 Table.

### *In vitro* phosphorylation assays

The experiment was conducted using 5 μg of purified recombinant substrate protein and 15 μg of purified nSTK to react in phosphorylation buffer (100 mM Tris HCl pH 8.0, 10 mM MgCl_2_, 25 mM NaCl, 100 mM ATP, and 1 mM DTT) at 37°C in a 50 μL system for 2 h. To accurately detect whether the recombinant proteins could be phosphorylated by nSTK *in vitro*, two control groups were used. In one group, only recombinant protein samples were added, and in the other group, only the nSTK protein was added. The experimental group contained both recombinant protein and nSTK protein. Protein loading buffer was added to all the samples immediately after the reaction, and the samples were boiled at 100°C for 10 min. The proteins were then separated by standard Tris-glycine‒SDS polyacrylamide gel electrophoresis (PAGE) gels and Phos-tag‒SDS polyacrylamide gels containing 100 μM phos-tag solution (ApexBio Technology LLC, Houston, TX, USA) and 200 μM MnCl_2_. After electrophoresis, the separated protein was observed by staining with Coomassie Brilliant Blue for 1 h and then decolorizing for a period of time.

### *In vivo* phosphorylation assays

WT SS2, *Δ*stk**, C*Δ*stk**, and FadR-T230A strain were cultured in THY liquid medium by shaking until the logarithmic phase and then washed three times with fresh sterile PBS. The bacteria were resuspended in 0.01 M PBS containing lysozyme (1 mg/mL), protease inhibitor cocktail (1:100), and phosphatase inhibitor cocktail (1:100). After being incubated at 37°C for 1 h, ultrasonic fragmentation treatment was performed on ice (20 min: 4 s on, 6 s off). After ultrasonic fragmentation, the samples were centrifuged at 12000 × *g* for 30 min at 4°C to remove cell debris. FadR polyclonal antibodies were added to the supernatant and incubated at 4°C for 2 h, followed by the addition of A/G agarose (Beyotime) and overnight rotation at 4°C for immunoprecipitation. After the agarose protein complex was washed three times with precooled PBS buffer, the agarose was boiled in 5 × SDS loading buffer for 10 min. The proteins were detected through Western blot analysis with FadR polyclonal antibodies and anti-phosphorylation (threonine) antibodies (Santa Cruz, USA). The experiment was performed three times.

### Stressor sensitivity assay

To explore the effects of stressors on the phosphorylation level of FadR, WT SS2 strains were grown in 40 mL of THY at 37°C until the OD_600_ reached ~0.6. The strains were subsequently washed three times with PBS, placed in pH 5.5 THY adjusted by hydrochloric acid, THY supplemented with 10 mM H_2_O_2_, and THY supplemented with 0.2 M NaCl, and incubated at 37°C for 30 min. Meanwhile, the survival rates of SS2 were calculated on the basis of the number of bacterial colonies before and after different treatment. Then, the strains were collected by centrifugation at 5000 × *g* and 4°C for 10 min. A protease inhibitor and phosphatase inhibitor were added to the strains, and the strains mixture was sonicated (power 300 w, ultrasonication for 4 s, 6 s interval, 10 min) in an ice bath and centrifuged at 12000 × *g* for 10 min at 4°C. Two microliters of FadR polyclonal antibody was added to the supernatant, and the mixture was incubated at 4°C for 2 h. Then, 50 μL of protein A/G agarose beads was added, and the mixture was incubated overnight at 4°C. The next day, the beads were collected by centrifugation at 4°C and 2500 × *g* for 5 min. After the precipitate was washed with precooled PBS three times, a certain amount of 5 × SDS‒PAGE loading buffer was added, and after boiling for 10 min, SDS‒PAGE gel electrophoresis and Western blot analysis were performed. For each experiment, at least three biological replicates were performed.

### Animal experiments

All 4‒6-week-old female mice were purchased from the Comparative Medicine Center of Yangzhou University (Yangzhou, China). To study the survival curves of the mice, the animals were randomly divided into different experimental groups, and intraperitoneally injected with WT SS2, *fadR* variants, *Δ*adi**, or FadR-T230E-C*adi* strains at a dose of 2 × 10^8^ CFUs. Sterile PBS was used as a blank control. Survival rates and clinical manifestations were closely monitored for each mouse within 7 days after inoculation with the bacteria. To investigate the colonization ability of different strains of bacteria in the internal organs of BALB/c mice, these bacteria were injected at a dose of 2 × 10^7^ CFUs. The bacterial loads in the blood, liver, spleen, lungs, and brain were measured 24 h after each strain was injected. To conduct pathological analysis of various tissues, the liver, spleen, lungs, and brain of infected BALB/c mice were first fixed and embedded in paraffin, then sectioned in paraffin, and finally stained with hematoxylin and eosin. All stained sections were scanned by white light through a panoramic scanner, and the lesions were observed. The blind method was used to evaluate the microscopic lesions in 5 random areas of each tissue slice, and scoring was performed as follows: 0, no lesions; 1, minimal; 2, mild; 3, moderate; and 4, severe.

### RNA-seq analysis

WT SS2 and *Δ*fadR** strains were cultured at 37°C until OD_600_ = 0.8. Each strain was cultured respectively in triplicate and then mixed in equal amounts [57]. Total RNA was extracted from bacteria using Total RNA Extraction Reagent (Vazyme Biotech Co., China) according to the manufacturer’s instructions. RNA-seq was performed at Genepioneer Biotechnologies (Nanjing, People’s Republic of China). Differential expression analysis between the two groups was conducted using the DESeq R package 1.18.0. The resulting *P* values were adjusted using Benjamini and Hochberg’s method to control the false discovery rate. Genes with an adjusted *P* value of ≤ 0.05 found by DESeq were assigned as differentially expressed.

### EMSA

A 6% nondenaturing polyacrylamide gel was used for electrophoresis to detect the binding of the FadR protein to the *adi* promoter. The online tool BProm program (SoftBerry) was used to predict the promoter sequence of *adi*, amplify the target fragment by PCR, and purify the obtained product with a gel electrophoresis kit. The negative control was a DNA fragments ~197 bp in length from the *enolase* promoter region. The purified DNA fragments were incubated with FadR or point mutant protein in binding buffer (10 mM Tris-base, 50 mM KCl, 5 mM MgCl_2_, 1 mM DTT, 0.05% Nonidet P-40, and 2.5% glycerol, pH 7.5) for 30 min at 37°C. Protein‒DNA complexes were added to a 6% nondenaturing polyacrylamide gel, which was then electrophoretically separated at 150 V in 0.5 × TBE buffer for 2.5 h and observed by ethidium bromide staining.

### RNA isolation and RT‒qPCR assays

Total RNA was extracted from bacteria with Total RNA Extraction Reagent (Vazyme Biotech Co., China). Bacterial cDNA samples were obtained with a HiScript II Q RT SuperMix Kit (Vazyme Biotech Co., China). All cDNA was used as a template for real-time quantitative PCR on a 7300 Real-Time PCR System (Applied Biosystems, Foster City, California, USA) with ChamQ Universal SYBR qPCR Master Mix (Vazyme Biotech Co., China). The primer sequences are listed in the S5 Table. The *gapdh* gene was used as an internal control, and the relative fold changes in transcript levels were calculated by the 2^-ΔΔCt^ method. The assays were performed in triplicate, and the qRT‒PCR experiments were repeated 3 times for each group.

### β-Galactosidase activity assays

The promoter of *adi* was amplified with the primers shown in the S5 Table. The obtained PCR products were fused with the empty plasmid pTCV-*lacZ* and then electroporated into WT SS2, *Δ*fadR**, FadR-T230A, and FadR-T230E. The determination of β-galactosidase activity was carried out by previously reported methods [58]. Each test was performed in triplicate and repeated at least three times.

### ChIP assay

After C*Δ*fadR**-*flag* was cultured to the logarithmic growth stage, it was washed three times with 0.01 M PBS, fixed at room temperature with 1% formaldehyde for 10 min, and finally crosslinked with 0.125 M glycine for 5 min. The bacterial cells were collected, centrifuged at 5000 × *g* at 4°C, washed three times with precooled 0.01 M PBS, and resuspended in cold lysis buffer. The genome was sonicated and fragmented into DNA fragments within the range of 0.5–1.0 kb. The sonicated sample was centrifuged at 12000 × *g* for 30 min at 4°C, and the supernatant was collected. Fifty microliters of the supernatant was retained. As an input, anti-flag antibody (Engibody, WI, USA) or negative mouse IgG (Beyotime, Shanghai, China) was added to the remaining supernatant for immunoprecipitation, and the mixture was incubated overnight on a rotating shaker at 4°C. Protein A/G beads (Beyotime) were added and incubated on a rotating shaker at 4°C for 2 h. The immunoprecipitation complexes were collected and washed three times each with ChIP washing buffer and TE solution, followed by elution with ChIP elution solution. All protein and RNA were removed by adding proteinase K and RNase (TAKARA, Dalian, China) to the eluent, and DNA fragments were recovered with a pure DNA fragment kit (Omega, USA). The input group and the recovered DNA fragment products were used as templates, and primers were designed on the basis of the *adi* promoter DNA sequence for PCR amplification and identification analysis. Each test was repeated at least three times.

### Western blots

Electrophoretically separated proteins were transferred onto polyvinylidene fluoride (PVDF, Millipore) membrane using a Semi-Dry Transfer Device (Bio-Rad) and run at 10 V for 20 min. The membrane was blocked in 5% non-fat milk or 1% bovine serum albumin for protein detection in TBST for 2 h at room temperature. The membranes were then incubated with primary antibodies at 4°C overnight. The primary antibodies used were as follows: Mouse anti-phosphotyrosine antibodies (Santa Cruz Biotechnology, Cat. No. sc-5267) at a 1:1000 dilution and mouse polyclonal anti-serum against FadR, ADI, GAPDH, and GroEL at a 1:1000 dilution.

The membranes were washed with TBST and then incubated with Anti-mouse IgG conjugated to HRP (1:5,000; Abmart, Cat. No. M21001S) at room temperature for 1 h. The membranes were incubated with ECL Femto-Detect Western Blotting Substrate (Engibody Biotechnology) and exposed using a ChemiDoc Touch Imaging System (Bio-Rad), and the resulting images were analyzed using Image Lab software. The immunoblots represent at least three independent replicates.

### Stressor sensitivity assay

The survival ability of bacteria under acid stress was determined according to the method of Roy S with slight modifications [59]. In general, SS2 was cultured to an OD_600_ of ~0.6. After centrifugation, the bacteria were washed 3 times in PBS and resuspended in 1 mL of PBS (pH 5.0) for 1 h at 37°C. The survival rates of bacteria were calculated on the basis of the number of bacterial colonies before and after acidic treatment. Each test was repeated three times.

### Intracellular survival assays

The intracellular survival assays of SS2 were performed with slight modifications compared with the method outlined in previous reports [60]. RAW264.7 cells were uniformly cultured in 24-well plates containing DMEM supplemented with 10% serum. When the cells were cultured to a density of 80%, bacteria were added at an initial multiplicity of infection multiple (MOI) of 10:1 and centrifuged at 400 × *g* at room temperature to promote sufficient direct contact between bacteria and cells. For the CQ group, RAW264.7 cells were treated with 40 μM CQ at the same time that bacterial infection. The cells were then cultured at 37°C and 5% CO_2_ for 1 h. After coculturing, the cells were washed three times with 0.01 M PBS, and then DMEM containing 100 µg/mL gentamicin and 10 µg/mL penicillin was added, followed by continuous cultivation for 1 h and 3 h. After cultivation, the cells were washed three times with PBS and then lysed with sterile water for 10 min. The lysate was continuously diluted and evenly dropped onto THY agar plates, followed by colony counting. The cell viability was calculated as CFU_3h_/CFU_1h _× 100%. All experiments were repeated 3 times, with 3 independent samples each time.

### Determining the intracellular pH in SS2

The calibration of the intracellular pH was carried out with using the manufacturer’s provided intracellular pH buffer calibration kit (Invitrogen, California, USA) as described previously [61]. The cells were treated with the intracellular pH calibration buffers by adding valinemycin (1 mM) and nigericin (1 mM) to ensure consistency in pH between the intracellular and extracellular environments, and then, the pH was measured by adding the intracellular fluorescent pH indicator BCECF-AM (Beyotime, Shanghai, China) and incubating at 37°C for 30 min. The fluorescence intensity was measured under 488 nm excitation and 535 nm excitation using a standard microplate reader. The calibration curve for SS2 was determined in the presence of various pH values ranging from 4.5–7.5 and was used in subsequent measurements to determine the intracellular pH in different strains of bacteria.

To measure the intracellular pH in SS2, the intracellular pH was measured with the indicator BCECF-AM as described previously. Bacteria were grown in THY media to an OD_600_ of ~0.6, washed with PBS 3 times, suspended in PBS (pH 7.6) with the indicator BCECF-AM and incubated at 37°C for 30 min. After 30 min, the fluorescence value was measured once, and then, the bacteria were switched from PBS medium with a pH value of 7.6 to PBS medium with a pH of 5.5 and incubated at 37°C for 1 h [62]. After incubation, the fluorescence value was measured once. Finally, the changes in the intracellular pH of the bacteria were compared. All spectra were measured for three biological replicates at the intracellular pH of each bacteria in 96-well black microplates (Beyotime). The experiment was repeated 3 times.

### Extraction and quantitative analysis of arginine

SS2 was cultured in THY medium to an OD_600_ of ~ 0.6, harvested by centrifugation (5 min; 5,000 × *g*; 4°C), washed 3 times, and subsequently resuspended in 0.01 M PBS at pH 5.5 for 1 h. Bacterial arginine concentrations were determined with an arginine assay kit (Solarbio, Beijing, China). The bacteria were disrupted by ultrasound in an ice bath (power 300 W, ultrasonication for 4 s, 6 s interval, 10 min) and centrifuged at 12000 × *g* for 10 min. A total of 800 μL of the supernatant was collected, after which 150 μL of extract solution 2 was slowly added, followed by blowing and mixing until no bubbles formed. The mixed mixture was centrifuged at 12000 × *g* for 10 min at 4°C, and the supernatant was removed for testing according to the instructions.

The different strains were added into the RAW264.7 cell holes at an MOI of 100:1 incubating for 1 h. The hole without strain was used as the control. The culture medium in these wells was replaced with DMEM containing gentamicin (100 µg/mL) and penicillin (10 µg/mL). The arginine content produced by RAW264.7 was determined using an arginine assay kit (Solarbio, China) after incubating for 3 h.

In addition, we incubated the recombinant protein of the ADI enzyme with the arginine standard at 37°C in reaction buffer (50 mM Tris, pH 7.6, 10 mM MgCl_2_, 1 mM DTT, and 5 mM ATP) and measured the arginine content every 5 min for a total of 30 min to better determine whether arginine could be broken down by ADI. Each assay was performed three times independently.

### Preparation of cell extracts and measurement of ammonia concentrations

The bacterial ammonia concentrations were determined with an ammonia assay kit (Abcam, UK). This reagent reacts with ammonia/ammonium and forms a fluorescent product. The fluorescence intensity (λ_ex/em_ = 360/450 nm) is proportional to the ammonia concentration in the sample, and the standard curve was should be established first. Similarly, when *S. suis* was cultured in THY medium to an OD_600_ of approximately 0.6, it was washed three times with PBS and then incubated at 37°C for 1 h in PBS (pH 5.5). After cultivation, 10 μL of the supernatant was directly centrifuged to determine the ammonia content. The remaining precipitate was washed three times with PBS and then sonicated (power 300 W, ultrasonication for 4 s, 6 s interval, 10 min) in an ice bath. After crushing, the mixture was centrifuged at 12000 × *g* for 10 min, and 10 μL of the supernatant was collected to measure the ammonia content. All ammonia content measurements were performed according to the manufacturer’s method.

### Determination of nitric-oxide production

The different strains were added into the RAW264.7 cell holes at an MOI of 100:1 incubating for 1 h. The hole without strain was used as the control. The culture medium in these wells was replaced with DMEM containing gentamicin (100 µg/mL) and penicillin (10 µg/mL). The NO content produced by RAW264.7 cells was determined using a micro NO content assay kit (Solarbio, China) after 24 h.

### ATR assays

The experimental method of ATR has been modified as described previously [16]. The bacteria were cultured to the logarithmic phase and then incubated at 37°C in THY with different pH values for 2 h to obtain the sublethal pH and lethal pH of WT SS2. For non-acid-induced conditions, bacterial cells were first grown in neutral THY at 37°C, and when cultures reached OD_600nm_ ~ 0.6, 100 μL aliquots were taken and added to 900 μL of THY (lethal pH) and incubated for 2 h at 37°C. In parallel, to determine survival under acidic-induced conditions, bacterial cells were grown in neutral THY until OD_600nm_ ~ 0.6, centrifuged at 5,000 *g* for 5 min, resuspended in THY (sublethal pH) and incubated for 2 h at 37°C. Then, 100 μL aliquots were taken and added to 900 μL of THY (lethal pH) and incubated for 2 h at 37°C. The survival rates of bacteria were calculated on the basis of the number of bacterial colonies before and after acidic treatment.

### Data analysis

GraphPad Prism software, version 8.0 (La Jolla, CA, USA), was used for data analysis in this study. All bar charts were analyzed with the mean ± standard deviation (SD) calculated from at least three biological replicates. All the data were analyzed with an unpaired *t* test (comparison of 2 groups) and one-way ANOVA or two-way ANOVA (comparison of survival curves) to analyze the statistical significance. A *P* value of < 0.05 was considered to indicate a significant difference.

## Supporting information

S1 Fig The mass spectrum of the peptide phosphorylated by LFNVSSphosITVIR shows that FadR is phosphorylated at Thr-230. (TIF)

S2 Fig The survival rate of SS2 under different environmental stresses. (A) Survival rates of WT SS2 after treatment with pH 5.5 THY for 30 min. (B) Survival rates of WT SS2 after treatment with 10 mM H_2_O_2_ THY for 30 min. (C) Survival rates of WT SS2 after treatment with 0.2 M NaCl THY for 30 min. Statistical analysis was performed by using an unpaired *t*-test (A, B, and C). ^ns^, *P* > 0.05.
(TIF)

S3 Fig Construction of *ΔfadR*, C*ΔfadR*, FadR-T230A, and FadR-T230E strains. (A) *ΔfadR* strain was identified by PCR with the primers *ΔfadR*-F1/*ΔfadR*-R2 (OUT) and FadR-F/FadR-R (IN). (B) C*ΔfadR* strain was identified by PCR with the primers pSET2-F/R (plasmid) and C*ΔfadR*-F/R (fragment). (C) FadR-T230A and FadR-T230E strains were subjected to PCR-based Sanger sequencing. (D) Growth curves of WT SS2, *ΔfadR*, C*ΔfadR*, FadR-T230A, and FadR-T230E strains in THY media were measured with a spectrophotometer at 600 nm.
(TIF)

S4 Fig Transcription and expression levels of FadR in WT SS2, *ΔfadR*, and C*ΔfadR* strains. (A) The *fadR* transcript levels in WT SS2, *ΔfadR*, and C*ΔfadR* strains were determined by RT‒qPCR. (B) The expression of FadR in WT SS2, *ΔfadR*, and C*ΔfadR* strains were detected by Western blotting. The band intensity relative to that of WT SS2 group was analyzed. The data shown represent three independent experiments and are presented as the means ± standard deviations. One-way ANOVA was used to test the significance of the data (A and B). ^ns^, *P* > 0.05; ^****^, *P* < 0.0001.
(TIF)

S5 Fig Pathological evaluation of mouse organs. (A-D) Pathological analysis of the liver (A), spleen (B), lung (C), and brain (D) by blinded assessment of H&E-stained sections. Statistical analysis was performed by using One-way ANOVA (A-D). ^*^, *P* < 0.05; ^**^, *P* < 0.01; ^****^, *P *< 0.0001.
(TIF)

S6 Fig Relative transcript levels of fadR in WT SS2 strain at different growth stages. Total RNA was extracted from SS2 at different OD_600_ values, and then reverse-transcribed into cDNA, followed by the determination of the relative transcription levels of FadR by RT‒qPCR. The data shown represent three independent experiments and are presented as the means ± standard deviations.
(TIF)

S7 Fig Construction of the Flag-tagged FadR strain. (A) WT SS2 and C*ΔfadR*-*flag* strains were assessed by Western blotting with an anti-FLAG antibody. (B) RAW264.7 cells were infected at an MOI of 10:1 with WT SS2, *ΔfadR*, and C*ΔfadR*-*flag* strains for 1 h. After 1 h of antibiotic sterilization (100 μg/mL gentamicin, 10 μg/mL penicillin), and sterile water was used to lyse the cells to release bacteria. Then bacteria were serial-diluted in PBS buffer and spread onto THY plates, incubated at 37°C for 16 h. The phagocytosis rate of each strain was calculated separately. The data shown represent three independent experiments and are presented as the means ± standard deviations. One-way ANOVA was used to test the significance of the data (B). ^ns^, *P* > 0.05; ^**^, *P* < 0.01.
(TIF)

S8 Fig The *adi* transcript levels in WT SS2 when treated in normal THY or acidified THY (pH 5.5). The data shown represent three independent experiments and are presented as the means ± standard deviations. ^***^, *P* < 0.001.
(TIF)

S9 Fig Growth of WT SS2, *Δadi*, C*Δadi*, FadR-T230E-C*adi*, ADI^Imp^, and ADI^Eno^ strains in THY medium measured with a spectrophotometer at 600 nm. The data shown represent three independent experiments and are presented as the means ± standard deviations.
(TIF)

S10 Fig The STK/FadR pathway modulates the acid tolerance response of SS2. (A) WT SS2 strain was treated with THY at different pH levels for 2 h, and the number of viable bacteria was subsequently determined by CFU plate counts. The bacterial survival rate was expressed as the ratio of the number of viable bacteria at 2 h to that at 0 h. (B) The ATRs of WT, *Δstk*, *ΔfadR*, FadR-T230A, and FadR-T230E strains were determined. To determine the survival percentage of bacterial strains, the non-induced cells (black bars) were directly exposed for 2 h at pH 4.5 (lethal pH) in THY medium, with the acid-induced cells (red bars) being previously incubated for 2 h at pH 6.0 (sub-lethal pH) in THY medium. After exposition to lethal pH, pneumococcal survival was determined by spreading dilutions in THY plates and incubating these at 37°C for 16 h. The data shown represent three independent experiments and are presented as the means ± standard deviations. Two-way ANOVA followed by Bonferroni’s multiple comparisons test (B). ^ns^, *P* > 0.05; ^****^, *P* < 0.0001.
(TIF)

S11 Fig Activity assessment of arginine deiminase *in vitro.* The recombinant protein ADI was co-incubated with arginine standard at 37°C in reaction buffer (50 mM Tris, pH 7.6, 10 mM MgCl_2_, 1 mM DTT, and 5 mM ATP) and measured the arginine content every 5 min for a total of 30 min. The data shown represent three independent experiments and are presented as the means ± standard deviations.
(TIF)

S12 Fig Establishment of the standard curve in this experiment. (A) A standard curve was established with the ammonia content on the horizontal axis and the fluorescence intensity on the vertical axis. (B) A standard curve was established with the pH value on the horizontal axis and the fluorescence intensity on the vertical axis. The data shown represent three independent experiments and are presented as the means ± standard deviations.
(TIF)

S13 Fig Detection of the effects of strains on acid stress. (A) Survival rates of WT SS2, *ΔfadR*, FadR-T230A, FadR-T230E, *Δadi*, and FadR-T230E-C*adi* strains after treatment with pH 5.5 PBS for 1 h. (B) *Δadi* strain was exposed to pH 5.5 PBS for 1 h, and the survival rate was measured at different time points. The data shown represent three independent experiments and are presented as the means ± standard deviations. One-way ANOVA was used to test the significance of the data (A). ^ns^, *P* > 0.05; ^*^, *P* < 0.05; ^***^, *P* < 0.001.
(TIF)

S14 Fig The regulatory effect of STK on *adi.* (A) WT SS2, *Δstk*, and C*Δstk* strains were treated with PBS at pH 5.0 for 1 h, and the number of viable bacteria was subsequently determined by CFU plate counts. The bacterial survival rate was expressed as the ratio of the number of viable bacteria at 1 h to that at 0 h. (B) The *adi* transcript levels in WT SS2, *Δstk*, and C*Δstk* strains were determined by RT‒qPCR in acidified THY (pH 5.5). (C) The expression of ADI in WT SS2, *Δstk*, and C*Δstk* strains were detected by Western blotting in acidified THY (pH 5.5). The band intensity relative to that of the WT SS2 group was analyzed. The data shown represent three independent experiments and are presented as the means ± standard deviations. One-way ANOVA was used to test the significance of the data (A-C). ^ns^, *P* > 0.05; ^**^, *P* < 0.01; ^***^, *P* < 0.001; ^****^, *P* < 0.0001.
(TIF)

S1 Table Phosphoproteomic results of FadR in WT SS2 and *Δstk* strains. (DOCX)

S2 Table Expression levels of genes in *ΔfadR* strains compared with those in WT SS2 strains. (XLSX)

S3 Table Differentially expressed genes in *ΔfadR* strains compared with those in WT SS2 strains. (DOCX)

S4 Table Strains and plasmids used in this study. (DOCX)

S5 Table Primers used in this study. (DOCX)

S1 Data Excel spreadsheet containing the numerical data and statistical analysis for Figure panels 1A, 1C, 2A-2C, 3A, 3B-3F, 4D-4E, 4G, 5A, 5B, 5C, 5D-5E, 6A-6H, S2A-2C, S3D, S4A-4B, S5A-5D, S6, S7B, S8, S9, S10A-10B, S11, S12A-12B, S13A-13B, and S14A-14C. (XLSX)
