# Supplementary figures and images for: STK-mediated FadR phosphorylation regulates the acid resistance and virulence of Streptococcus suis
Source: PLoS Pathog. 2025 Sep 25;21(9):e1013534. doi: 10.1371/journal.ppat.1013534 (PMC12463286; doi:10.1371/journal.ppat.1013534)

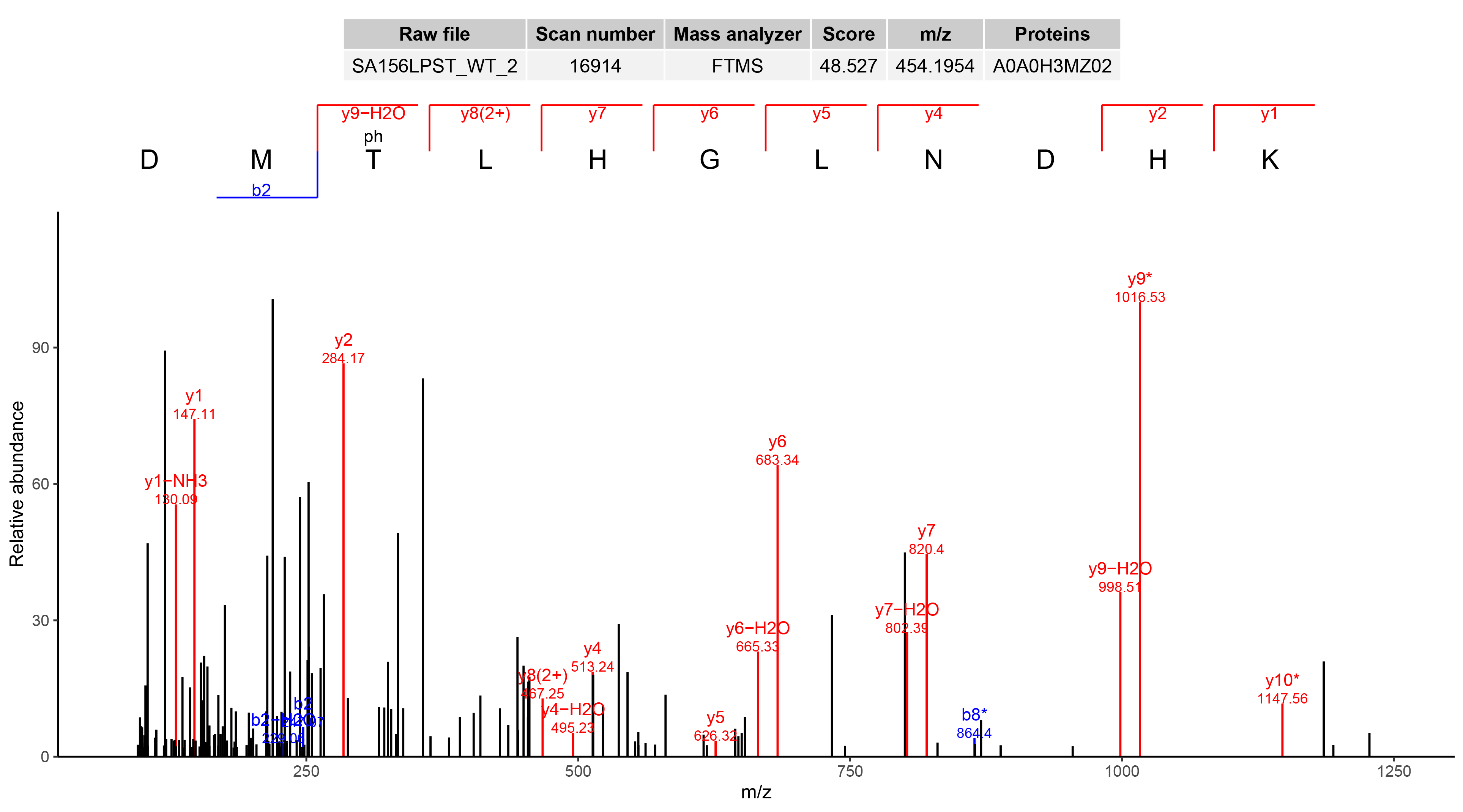

Supplement: S1 Fig — (TIF) [file ppat.1013534.s001.tif]

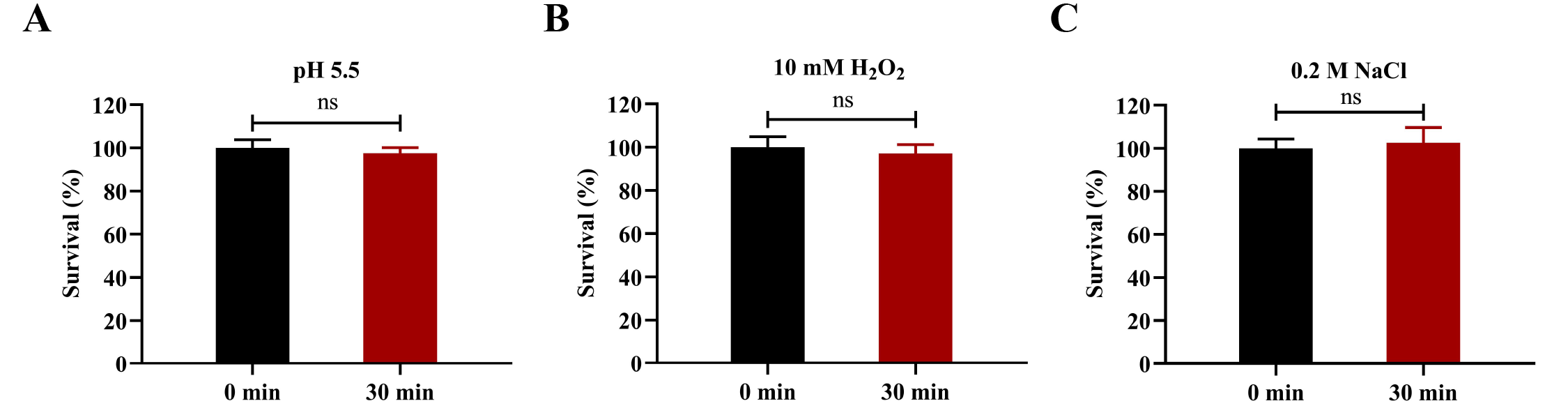

Supplement: S2 Fig — (A) Survival rates of WT SS2 after treatment with pH 5.5 THY for 30 min. (B) Survival rates of WT SS2 after treatment with 10 mM H2O2 THY for 30 min. (C) Survival rates of WT SS2 after treatment with 0.2 M NaCl THY for 30 min. Statistical analysis was performed by using an unpaired t-test (A, B, and C). ns, P > 0.05. (TIF) [file ppat.1013534.s002.tif]

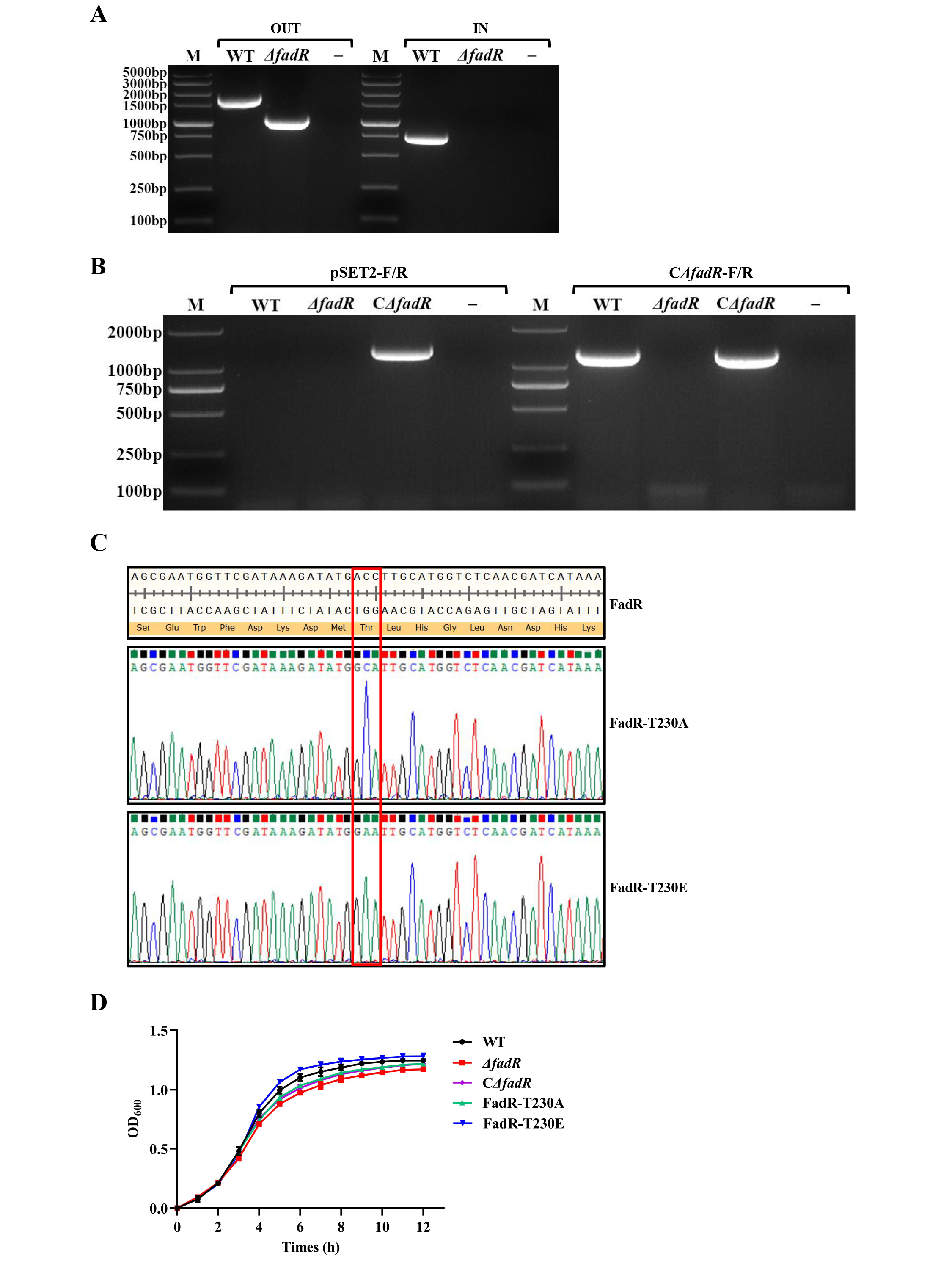

Supplement: S3 Fig — (A) ΔfadR strain was identified by PCR with the primers ΔfadR-F1/ΔfadR-R2 (OUT) and FadR-F/FadR-R (IN). (B) CΔfadR strain was identified by PCR with the primers pSET2-F/R (plasmid) and CΔfadR-F/R (fragment). (C) FadR-T230A and FadR-T230E strains were subjected to PCR-based Sanger sequencing. (D) Growth curves of WT SS2, ΔfadR, CΔfadR, FadR-T230A, and FadR-T230E strains in THY media were measured with a spectrophotometer at 600 nm. (TIF) [file ppat.1013534.s003.tif]

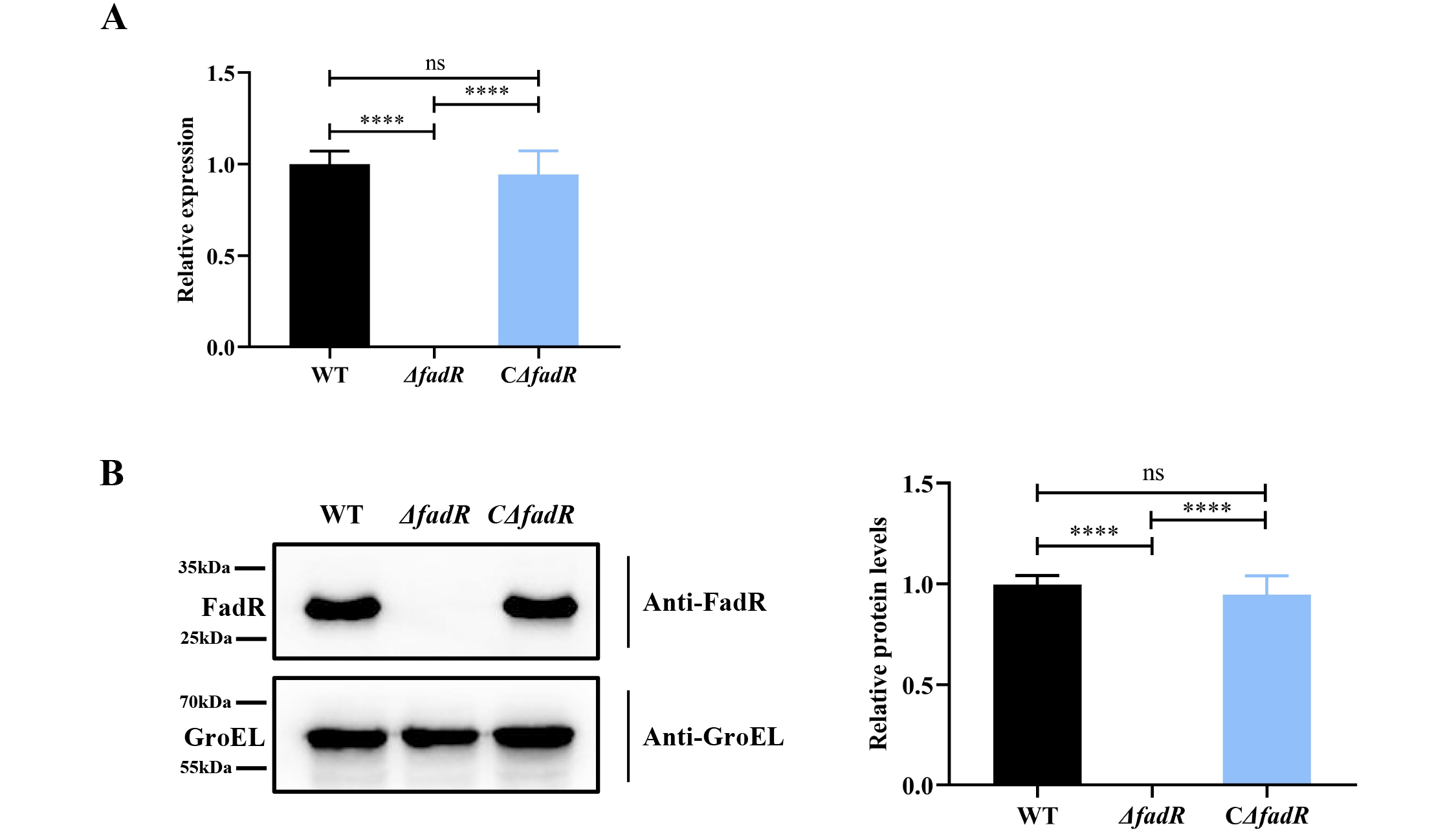

Supplement: S4 Fig — (A) The fadR transcript levels in WT SS2, ΔfadR, and CΔfadR strains were determined by RT‒qPCR. (B) The expression of FadR in WT SS2, ΔfadR, and CΔfadR strains were detected by Western blotting. The band intensity relative to that of WT SS2 group was analyzed. The data shown represent three independent experiments and are presented as the means ± standard deviations. One-way ANOVA was used to test the significance of the data (A and B). ns, P > 0.05; ****, P < 0.0001. (TIF) [file ppat.1013534.s004.tif]

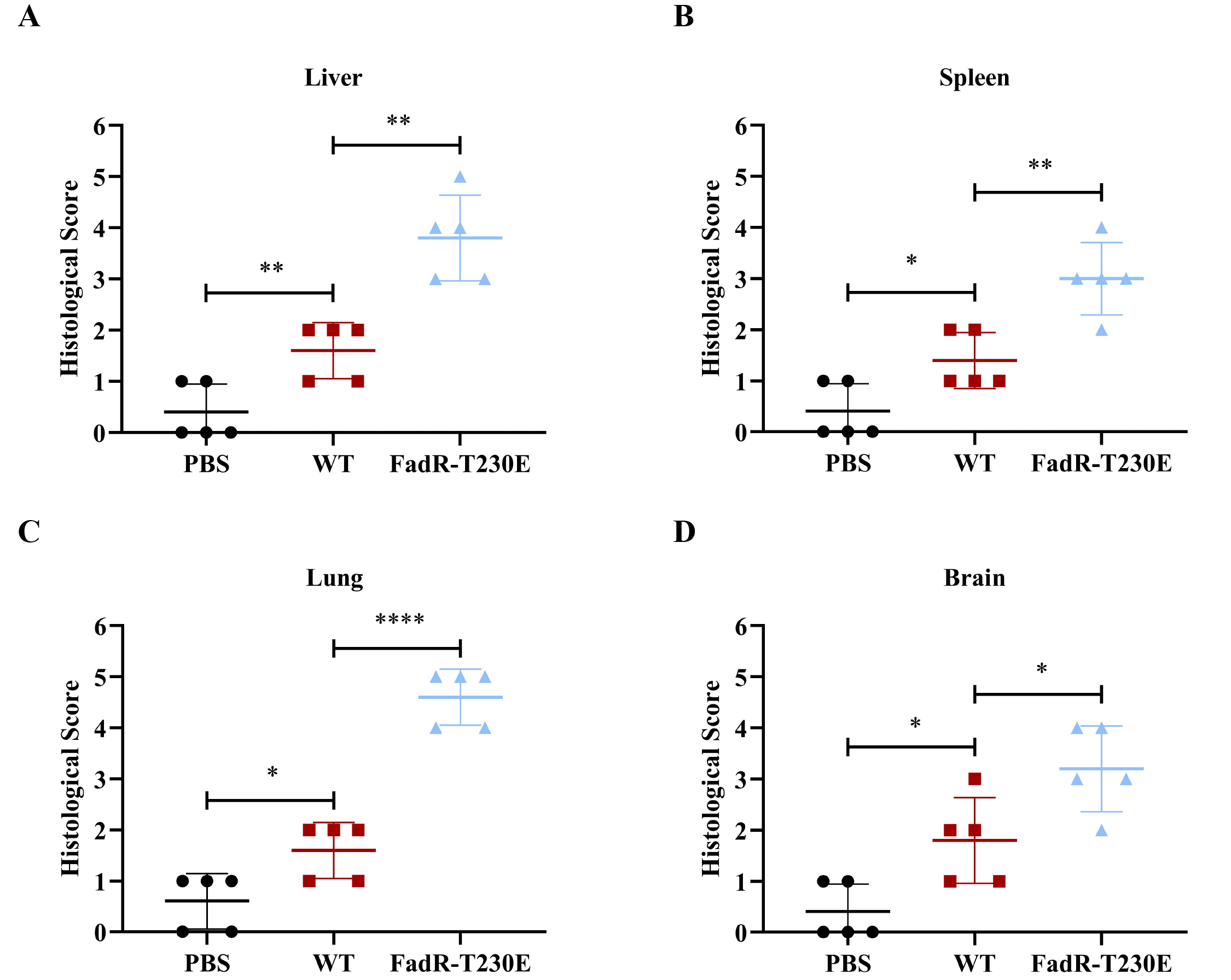

Supplement: S5 Fig — (A-D) Pathological analysis of the liver (A), spleen (B), lung (C), and brain (D) by blinded assessment of H&E-stained sections. Statistical analysis was performed by using One-way ANOVA (A-D). *, P < 0.05; **, P < 0.01; ****, P < 0.0001. (TIF) [file ppat.1013534.s005.tif]

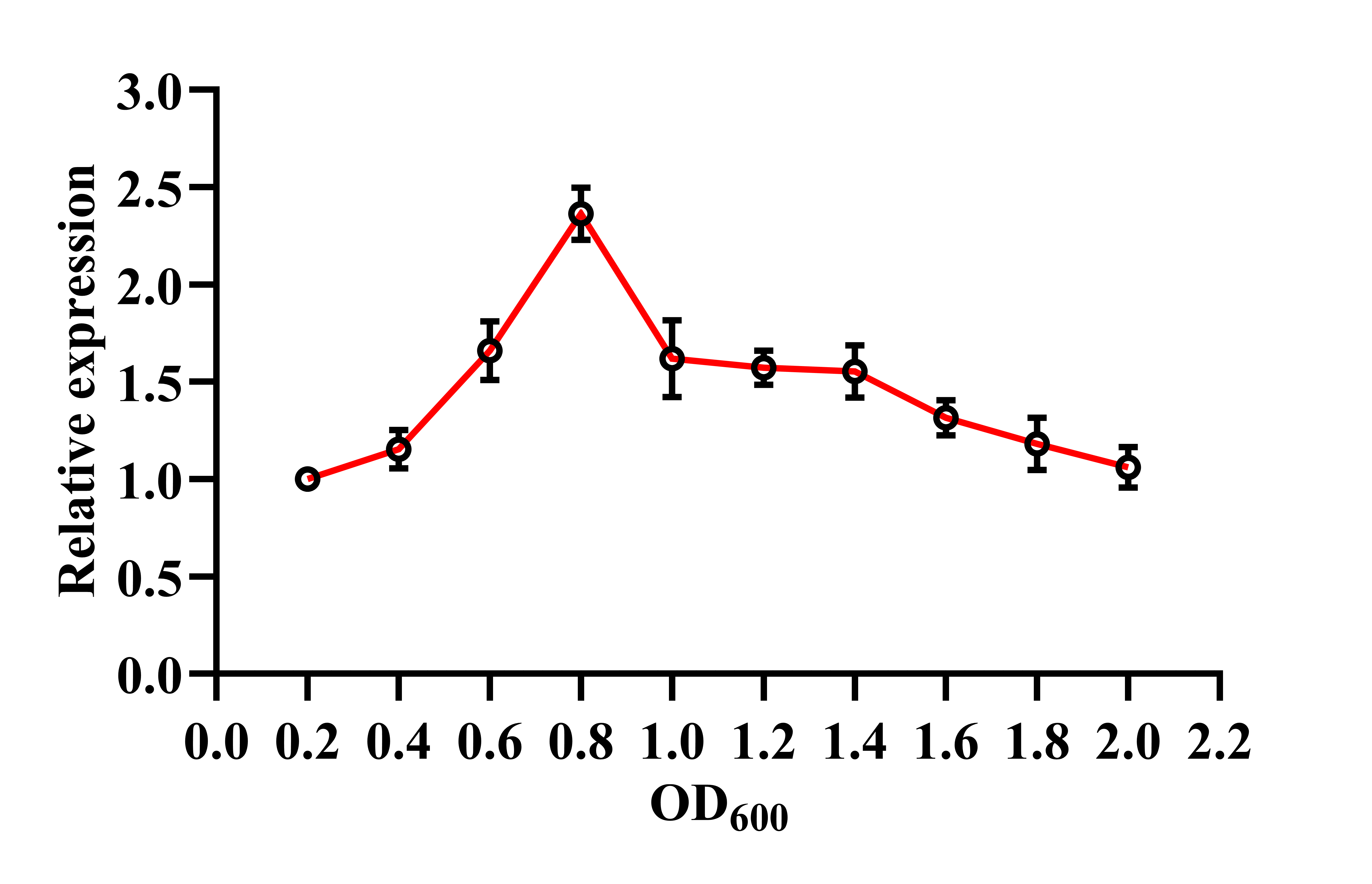

Supplement: S6 Fig — Total RNA was extracted from SS2 at different OD600 values, and then reverse-transcribed into cDNA, followed by the determination of the relative transcription levels of FadR by RT‒qPCR. The data shown represent three independent experiments and are presented as the means ± standard deviations. (TIF) [file ppat.1013534.s006.tif]

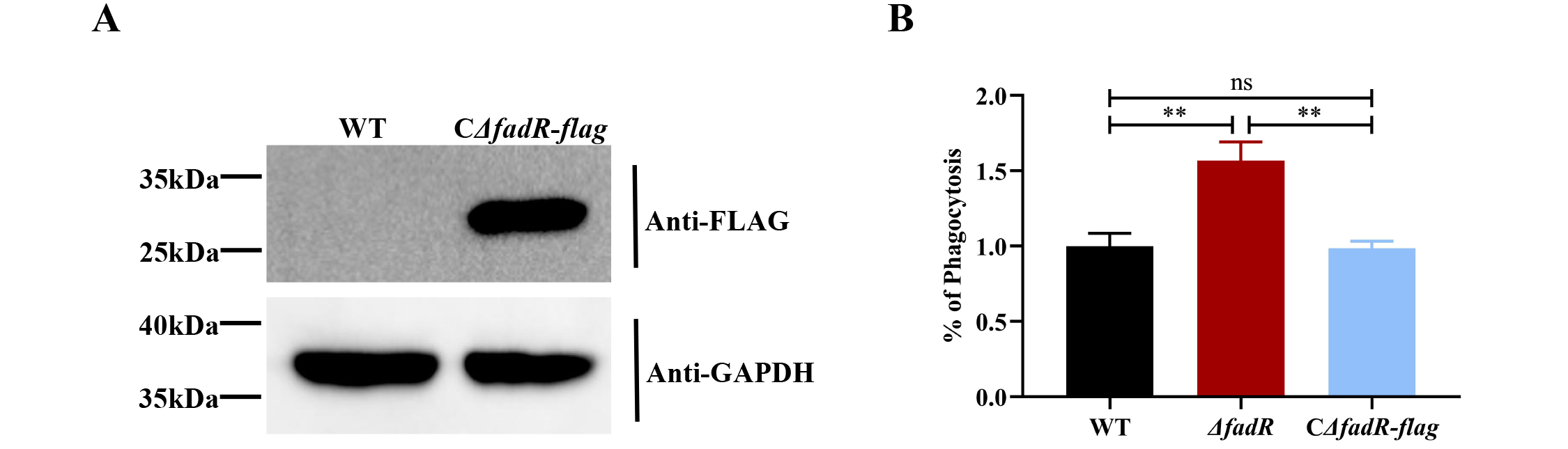

Supplement: S7 Fig — (A) WT SS2 and CΔfadR-flag strains were assessed by Western blotting with an anti-FLAG antibody. (B) RAW264.7 cells were infected at an MOI of 10:1 with WT SS2, ΔfadR, and CΔfadR-flag strains for 1 h. After 1 h of antibiotic sterilization (100 μg/mL gentamicin, 10 μg/mL penicillin), and sterile water was used to lyse the cells to release bacteria. Then bacteria were serial-diluted in PBS buffer and spread onto THY plates, incubated at 37°C for 16 h. The phagocytosis rate of each strain was calculated separately. The data shown represent three independent experiments and are presented as the means ± standard deviations. One-way ANOVA was used to test the significance of the data (B). ns, P > 0.05; **, P < 0.01. (TIF) [file ppat.1013534.s007.tif]

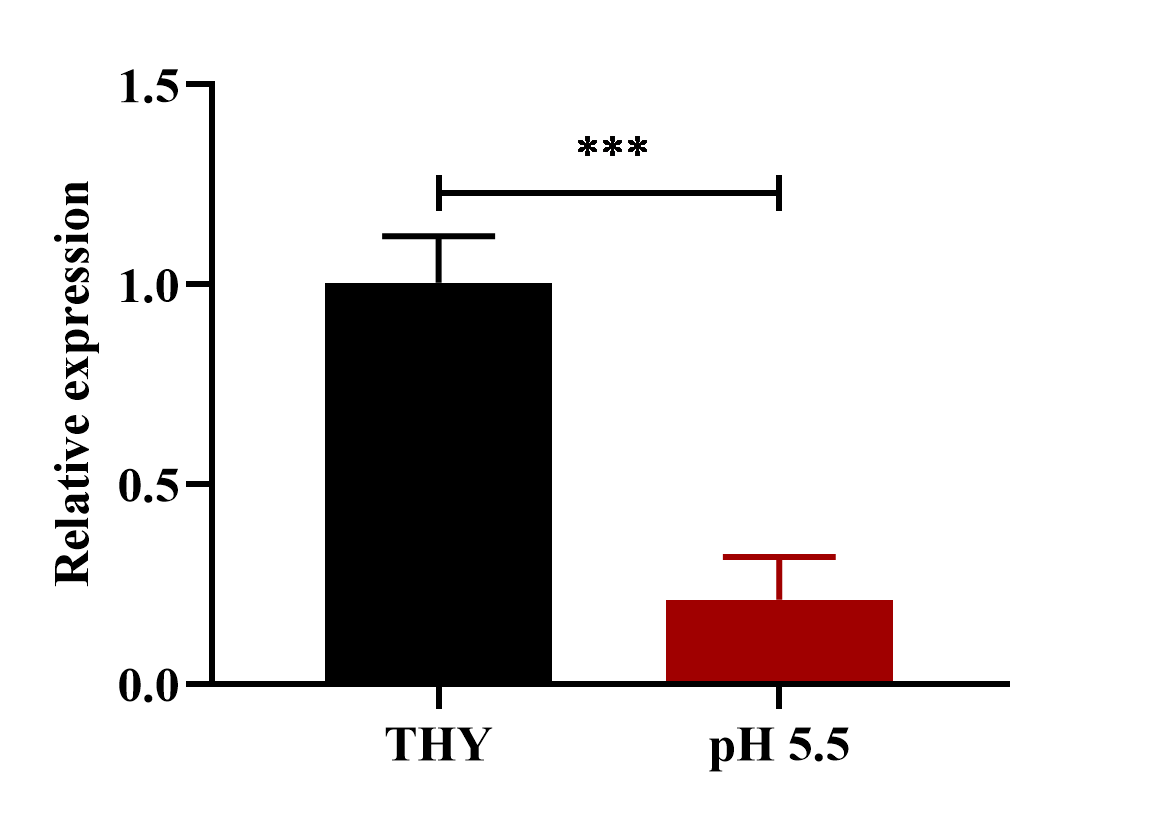

Supplement: S8 Fig — The data shown represent three independent experiments and are presented as the means ± standard deviations. ***, P < 0.001. (TIF) [file ppat.1013534.s008.tif]

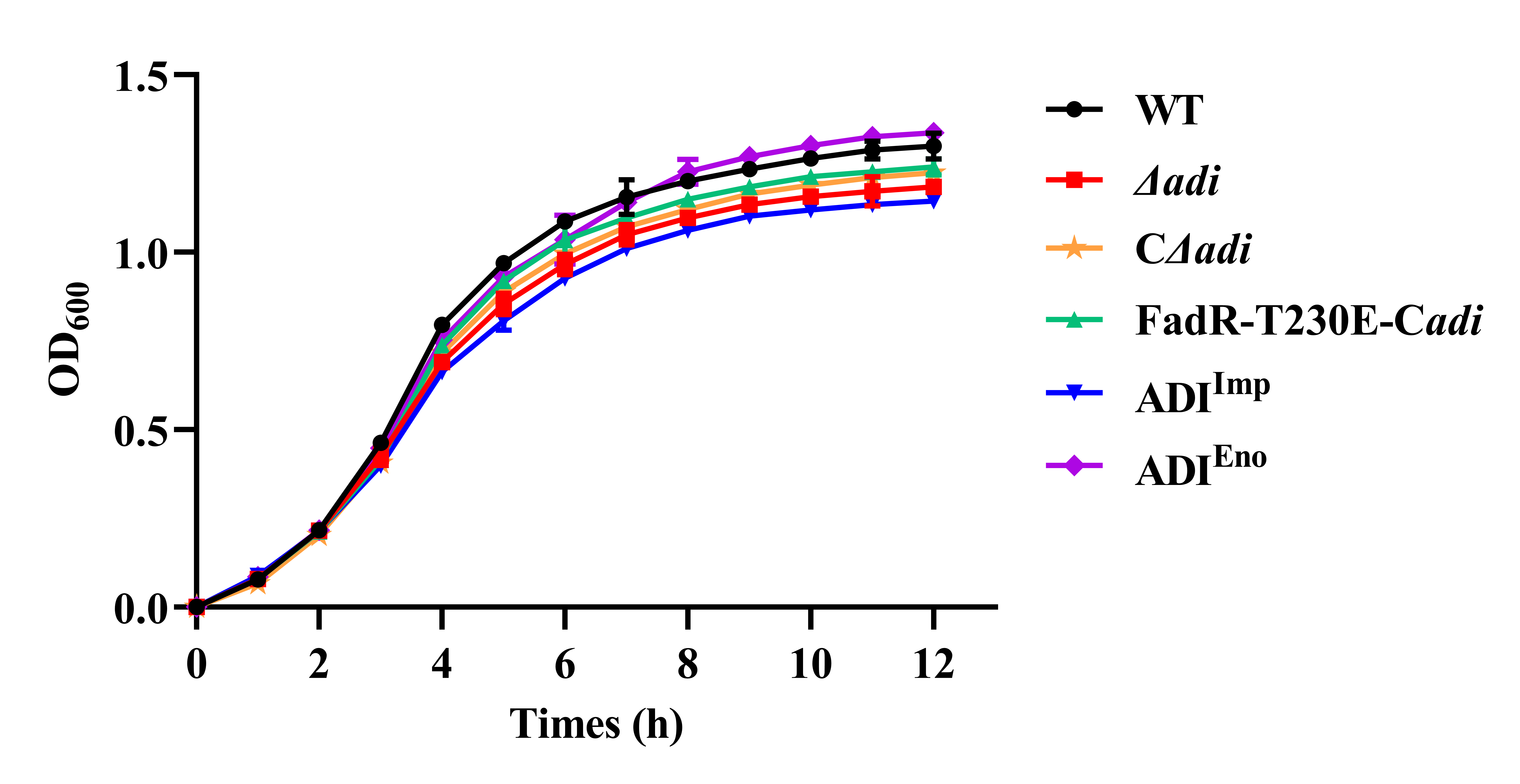

Supplement: S9 Fig — The data shown represent three independent experiments and are presented as the means ± standard deviations. (TIF) [file ppat.1013534.s009.tif]

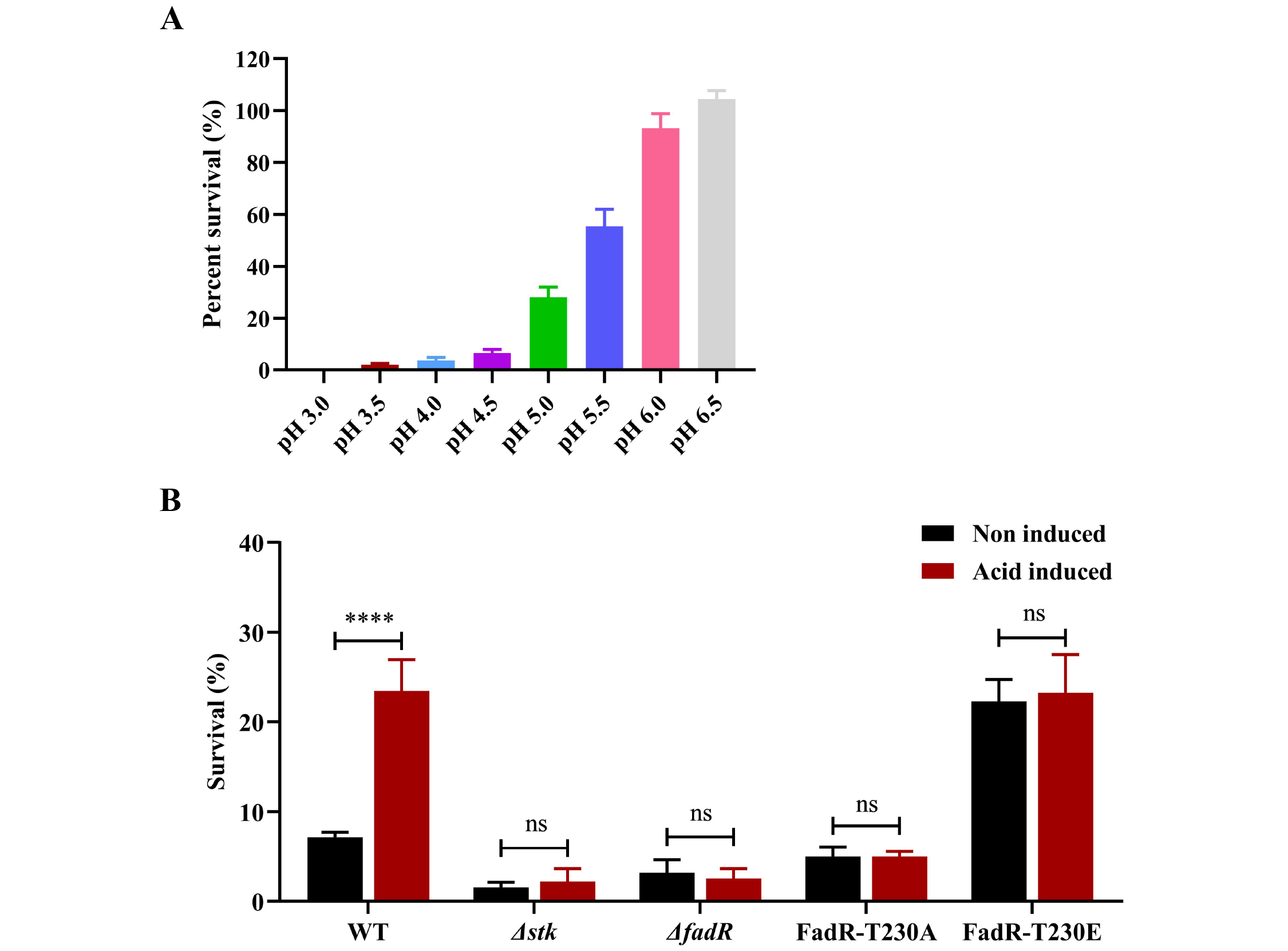

Supplement: S10 Fig — (A) WT SS2 strain was treated with THY at different pH levels for 2 h, and the number of viable bacteria was subsequently determined by CFU plate counts. The bacterial survival rate was expressed as the ratio of the number of viable bacteria at 2 h to that at 0 h. (B) The ATRs of WT, Δstk, ΔfadR, FadR-T230A, and FadR-T230E strains were determined. To determine the survival percentage of bacterial strains, the non-induced cells (black bars) were directly exposed for 2 h at pH 4.5 (lethal pH) in THY medium, with the acid-induced cells (red bars) being previously incubated for 2 h at pH 6.0 (sub-lethal pH) in THY medium. After exposition to lethal pH, pneumococcal survival was determined by spreading dilutions in THY plates and incubating these at 37°C for 16 h. The data shown represent three independent experiments and are presented as the means ± standard deviations. Two-way ANOVA followed by Bonferroni’s multiple comparisons test (B). ns, P > 0.05; ****, P < 0.0001. (TIF) [file ppat.1013534.s010.tif]

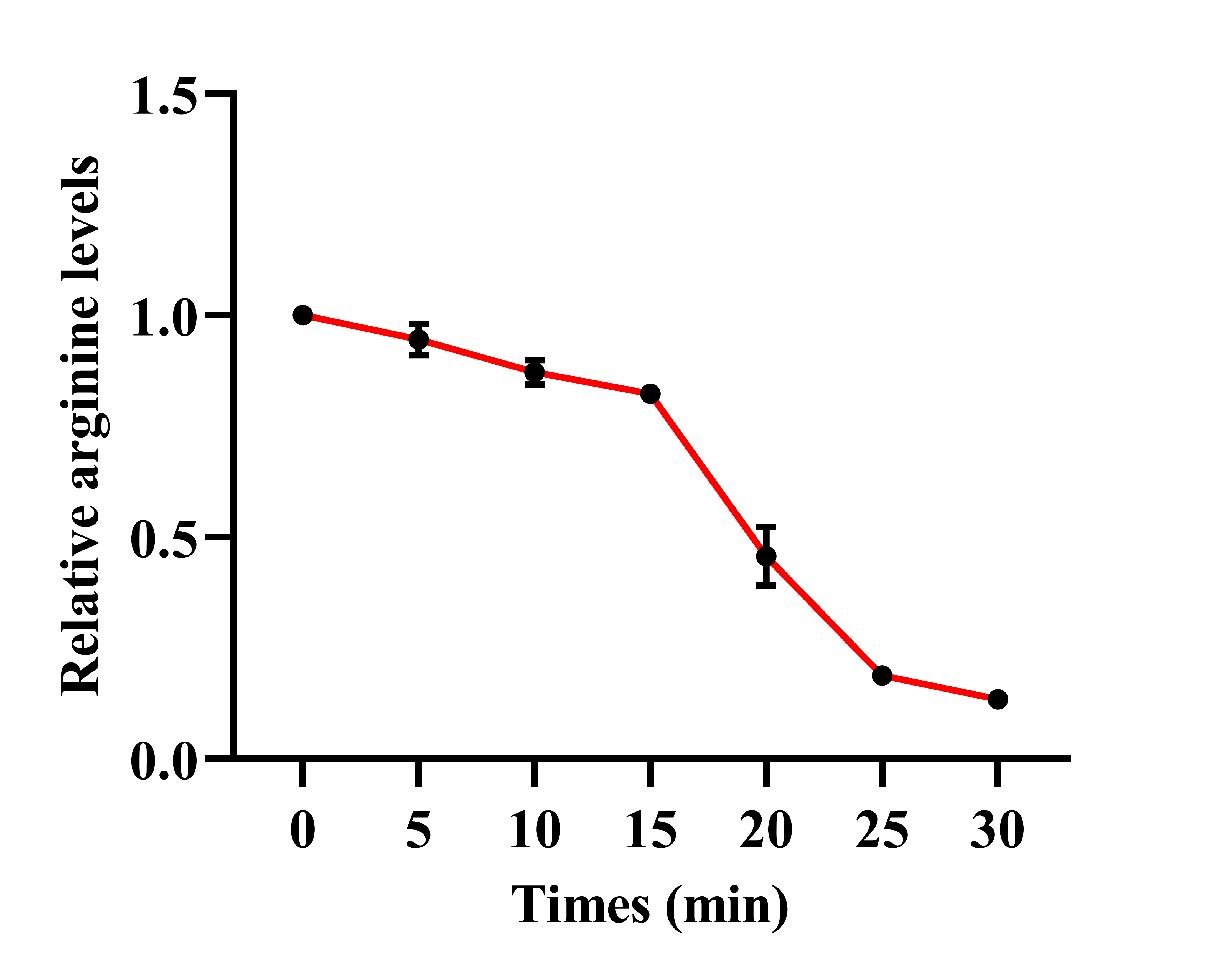

Supplement: S11 Fig — The recombinant protein ADI was co-incubated with arginine standard at 37°C in reaction buffer (50 mM Tris, pH 7.6, 10 mM MgCl2, 1 mM DTT, and 5 mM ATP) and measured the arginine content every 5 min for a total of 30 min. The data shown represent three independent experiments and are presented as the means ± standard deviations. (TIF) [file ppat.1013534.s011.tif]

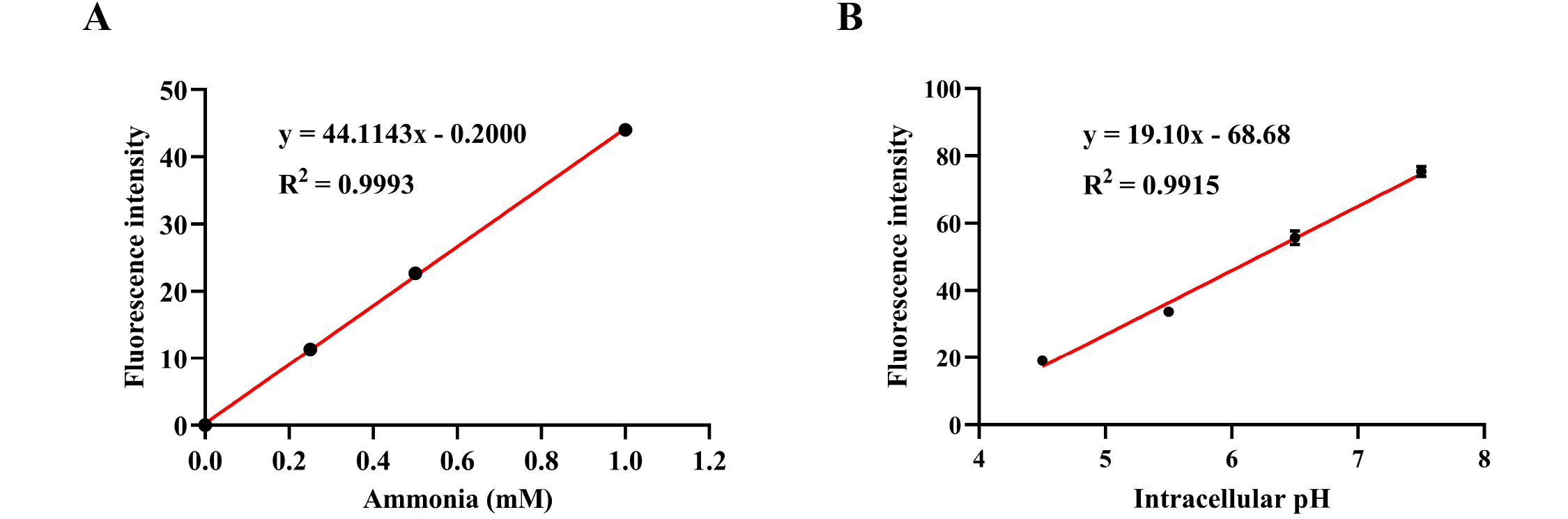

Supplement: S12 Fig — (A) A standard curve was established with the ammonia content on the horizontal axis and the fluorescence intensity on the vertical axis. (B) A standard curve was established with the pH value on the horizontal axis and the fluorescence intensity on the vertical axis. The data shown represent three independent experiments and are presented as the means ± standard deviations. (TIF) [file ppat.1013534.s012.tif]

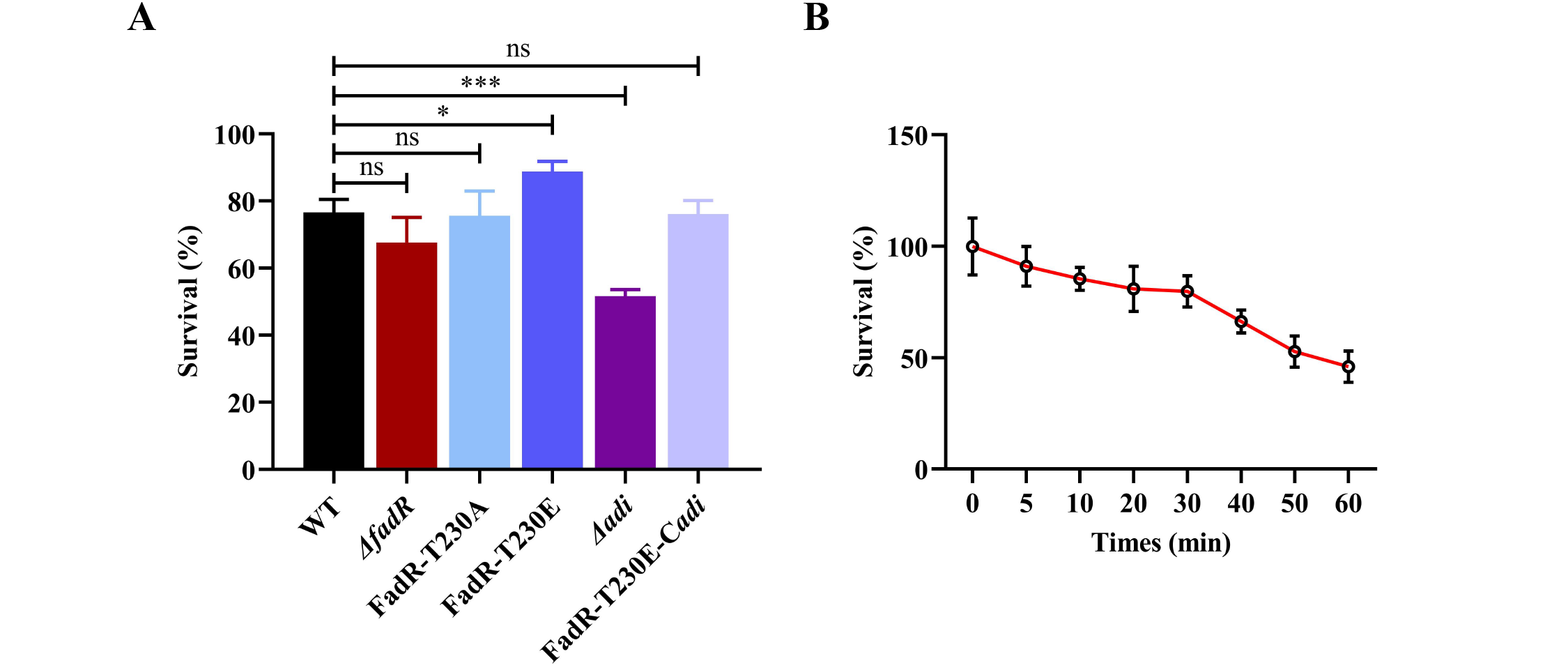

Supplement: S13 Fig — (A) Survival rates of WT SS2, ΔfadR, FadR-T230A, FadR-T230E, Δadi, and FadR-T230E-Cadi strains after treatment with pH 5.5 PBS for 1 h. (B) Δadi strain was exposed to pH 5.5 PBS for 1 h, and the survival rate was measured at different time points. The data shown represent three independent experiments and are presented as the means ± standard deviations. One-way ANOVA was used to test the significance of the data (A). ns, P > 0.05; *, P < 0.05; ***, P < 0.001. (TIF) [file ppat.1013534.s013.tif]

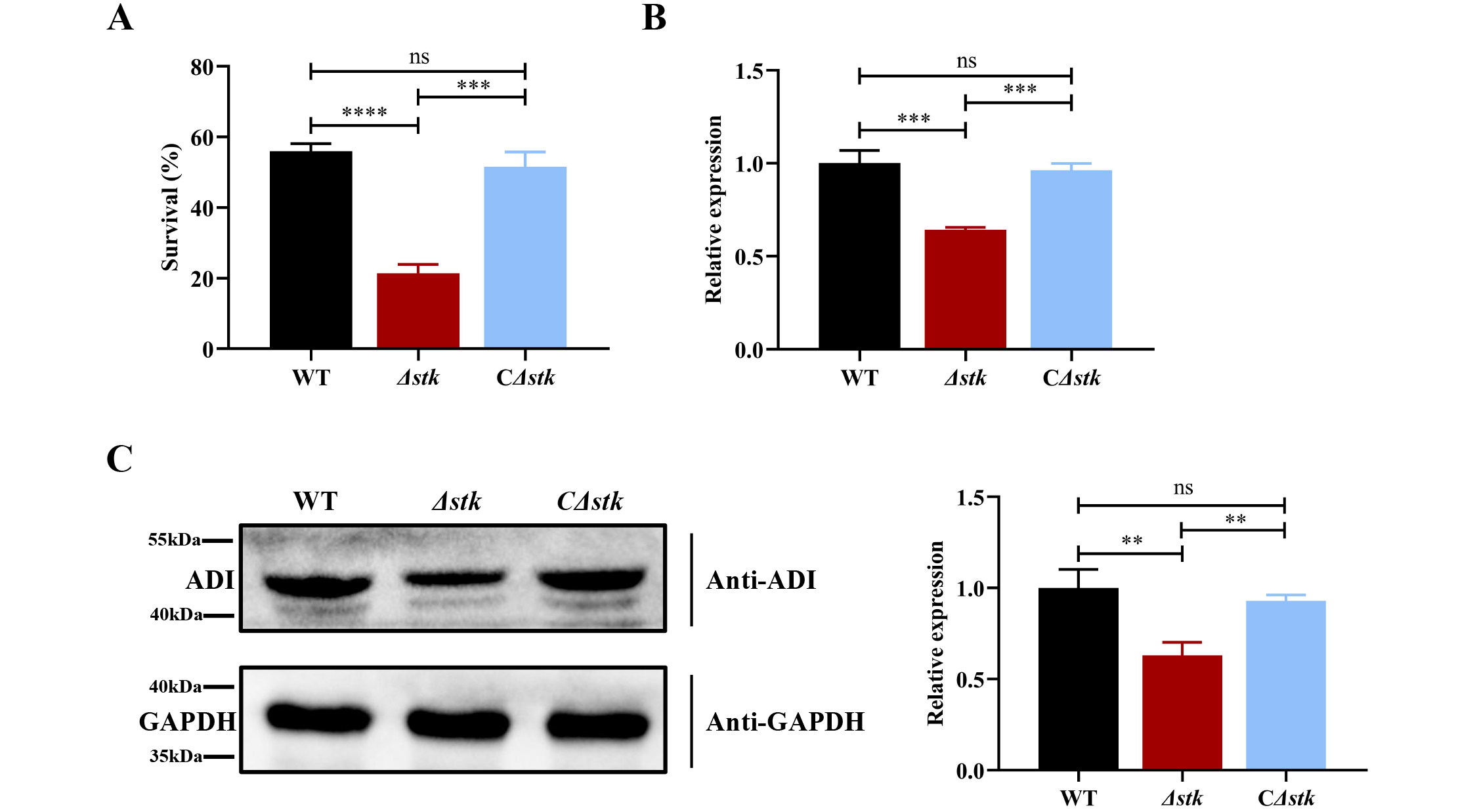

Supplement: S14 Fig — (A) WT SS2, Δstk, and CΔstk strains were treated with PBS at pH 5.0 for 1 h, and the number of viable bacteria was subsequently determined by CFU plate counts. The bacterial survival rate was expressed as the ratio of the number of viable bacteria at 1 h to that at 0 h. (B) The adi transcript levels in WT SS2, Δstk, and CΔstk strains were determined by RT‒qPCR in acidified THY (pH 5.5). (C) The expression of ADI in WT SS2, Δstk, and CΔstk strains were detected by Western blotting in acidified THY (pH 5.5). The band intensity relative to that of the WT SS2 group was analyzed. The data shown represent three independent experiments and are presented as the means ± standard deviations. One-way ANOVA was used to test the significance of the data (A-C). ns, P > 0.05; **, P < 0.01; ***, P < 0.001; ****, P < 0.0001. (TIF) [file ppat.1013534.s014.tif]
