# Supplementary material for: STK-mediated FadR phosphorylation regulates the acid resistance and virulence of Streptococcus suis
Source: PLoS Pathog. 2025 Sep 25;21(9):e1013534. doi: 10.1371/journal.ppat.1013534 (PMC12463286; doi:10.1371/journal.ppat.1013534)
Supplement: S1 Table — (DOCX) [file ppat.1013534.s015.docx]

**S1 Table.** Phosphoproteomics results of FadR in WT SS2 and *Δstk*.

| Protein accession | A0A0H3MZ02 |
| --- | --- |
| Position | 230 |
| Amino acid | T |
| Protein description | GntR family regulatory protein OS=*Streptococcus suis* (strain BM407) OX=568814 GN=SSUBM407_0781 PE=4 SV=1 |
| Gene name | SSUBM407_0781 |
| Localization probability | 1 |
| PEP | 0.0016637 |
| Score | 48.527 |
| Charge | 3 |
| Modified sequence | DMT(1)LHGLNDHK |
| Mass error [ppm] | -0.53707 |
| Stk_1 |  |
| Stk_2 |  |
| Stk_3 |  |
| WT_1 |  |
| WT_2 | 0.958 |
| WT_3 | 1.1 |
| Stk/WT Ratio |  |
| Stk/WT P value |  |
| Subcellular localizatio | Cytoplasmic |
