# Supplementary material for: STK-mediated FadR phosphorylation regulates the acid resistance and virulence of Streptococcus suis
Source: PLoS Pathog. 2025 Sep 25;21(9):e1013534. doi: 10.1371/journal.ppat.1013534 (PMC12463286; doi:10.1371/journal.ppat.1013534)
Supplement: S3 Table — (DOCX) [file ppat.1013534.s017.docx]

**S3 Table.** Expression levels of genes in *ΔfadR* compared to WT SS2.

| Code for ORF | log2Fold Change  (*ΔfadR*/WT) | *p*-value | Functional annotation |
| --- | --- | --- | --- |
| ZY05719_05540 | 5.694419985 | 4.69E-24 | glycoside hydrolase family 3 N-terminal domain-containing protein |
| ZY05719_05545 | 5.417596008 | 2.07E-22 | glycoside hydrolase family 3 N-terminal domain-containing protein |
| ZY05719_05550 | 4.982235286 | 5.50E-20 | HAD family hydrolase |
| ZY05719_05555 | 4.890989139 | 1.78E-19 | HAD family hydrolase |
| ZY05719_05560 | 4.250950633 | 6.07E-16 | mannonate dehydratase |
| ZY05719_05565 | 3.556301925 | 2.50E-12 | glucuronate isomerase |
| ZY05719_05570 | 3.198182811 | 1.81E-10 | bifunctional 4-hydroxy-2-oxoglutarate aldolase/2-dehydro-3-deoxy-phosphogluconate aldolase |
| ZY05719_03720 | 3.009407608 | 2.41E-09 | tRNA-Tyr |
| ZY05719_02130 | 2.940094185 | 3.62E-09 | tRNA-Ala |
| ZY05719_01705 | 2.932508154 | 3.75E-08 | tRNA-Asn |
| ZY05719_00130 | 2.923962858 | 3.36E-09 | tRNA-Gly |
| ZY05719_02170 | 2.904471032 | 4.09E-09 | tRNA-Gly |
| ZY05719_05765 | 2.83624763 | 8.19E-09 | aconitate hydratase AcnA |
| ZY05719_00125 | 2.826495835 | 9.36E-09 | tRNA-Thr |
| ZY05719_05580 | 2.783946426 | 5.38E-08 | beta-glucuronidase |
| ZY05719_02165 | 2.73296654 | 2.53E-08 | tRNA-Thr |
| ZY05719_00090 | 2.722277035 | 3.35E-08 | tRNA-Ala |
| ZY05719_01690 | 2.688203659 | 6.30E-08 | tRNA-Ala |
| ZY05719_00135 | 2.668308013 | 4.92E-08 | tRNA-Leu |
| ZY05719_00120 | 2.647834456 | 5.98E-08 | tRNA-Leu |
| ZY05719_00580 | 2.644834064 | 9.72E-08 | tRNA-Ala |
| ZY05719_02175 | 2.644772996 | 6.36E-08 | tRNA-Leu |
| ZY05719_00910 | 2.592292335 | 1.01E-07 | MerR family transcriptional regulator |
| ZY05719_00140 | 2.573779548 | 1.32E-07 | tRNA-Arg |
| ZY05719_06500 | 2.482297334 | 3.05E-07 | amino acid ABC transporter ATP-binding protein |
| ZY05719_02185 | 2.466347104 | 8.11E-07 | tRNA-Pro |
| ZY05719_02160 | 2.451334142 | 4.27E-07 | tRNA-Leu |
| ZY05719_00115 | 2.430747511 | 5.32E-07 | tRNA-Lys |
| ZY05719_00145 | 2.414057228 | 6.15E-07 | tRNA-Pro |
| ZY05719_02180 | 2.404588451 | 7.51E-07 | tRNA-Arg |
| ZY05719_08565 | 2.368433246 | 9.21E-07 | enoyl-[acyl-carrier-protein] reductase FabK |
| ZY05719_02155 | 2.3292866 | 1.39E-06 | tRNA-Lys |
| ZY05719_00110 | 2.306300918 | 1.73E-06 | tRNA-Asp |
| ZY05719_03715 | 2.26283992 | 3.05E-06 | DUF2969 domain-containing protein |
| ZY05719_00150 | 2.254129873 | 2.80E-06 | tRNA-Met |
| ZY05719_02150 | 2.24143831 | 3.27E-06 | tRNA-Asp |
| ZY05719_00655 | 2.231345481 | 5.15E-06 | tyrosine-type recombinase/integrase |
| ZY05719_05760 | 2.218754645 | 3.96E-06 | citrate synthase |
| ZY05719_09670 | 2.21861879 | 3.82E-06 | MarR family transcriptional regulator |
| ZY05719_00915 | 2.196398116 | 4.64E-06 | type I glutamate--ammonia ligase |
| ZY05719_00105 | 2.175525032 | 5.83E-06 | tRNA-Val |
| ZY05719_02145 | 2.138590641 | 8.46E-06 | tRNA-Val |
| ZY05719_00155 | 2.128943497 | 8.77E-06 | tRNA-Ile |
| ZY05719_01670 | 2.114622006 | 1.49E-05 | tRNA-Leu |
| ZY05719_00650 | 2.07995332 | 1.41E-05 | tRNA-Leu |
| ZY05719_00610 | 2.059658562 | 1.62E-05 | tRNA-Glu |
| ZY05719_06505 | 2.058048042 | 1.62E-05 | transporter substrate-binding domain-containing protein |
| ZY05719_00615 | 2.038467903 | 1.98E-05 | tRNA-Ser |
| ZY05719_00100 | 2.005621137 | 2.94E-05 | 5S ribosomal RNA |
| ZY05719_02140 | 1.998891885 | 4.80E-05 | 5S ribosomal RNA |
| ZY05719_00100 | 1.998891885 | 4.80E-05 | 5S ribosomal RNA |
| ZY05719_02685 | 1.986941013 | 2.99E-05 | glutamine--fructose-6-phosphate transaminase (isomerizing) |
| ZY05719_05590 | 1.969337319 | 6.04E-05 | MFS transporter |
| ZY05719_00605 | 1.956050252 | 3.96E-05 | tRNA-Ile |
| ZY05719_03665 | 1.936762652 | 4.60E-05 | glycerol-3-phosphate 1-O-acyltransferase PlsY |
| ZY05719_00160 | 1.900833732 | 6.34E-05 | tRNA-Ser |
| ZY05719_00600 | 1.900057258 | 6.35E-05 | tRNA-Gly |
| ZY05719_08585 | 1.892507001 | 6.67E-05 | enoyl-CoA hydratase |
| ZY05719_06715 | 1.876982071 | 0.000170866 | tRNA-Thr |
| ZY05719_00620 | 1.874314476 | 8.15E-05 | tRNA-Met |
| ZY05719_00595 | 1.860844285 | 8.80E-05 | tRNA-Val |
| ZY05719_08560 | 1.79704434 | 0.000145601 | ACP S-malonyltransferase |
| ZY05719_00590 | 1.787511224 | 0.000164696 | 5S ribosomal RNA |
| ZY05719_05755 | 1.780671857 | 0.00016954 | NADP-dependent isocitrate dehydrogenase |
| ZY05719_00645 | 1.776935941 | 0.000176038 | tRNA-Gln |
| ZY05719_02500 | 1.757139732 | 0.000201123 | biotin transporter BioY |
| ZY05719_00625 | 1.72949391 | 0.000260714 | tRNA-Phe |
| ZY05719_00635 | 1.687916869 | 0.000352757 | tRNA-Trp |
| ZY05719_00640 | 1.684968823 | 0.000360107 | tRNA-His |
| ZY05719_08580 | 1.664205667 | 0.000413076 | MarR family transcriptional regulator |
| ZY05719_00165 | 1.651142309 | 0.000465745 | tRNA-Met |
| ZY05719_03350 | 1.650685094 | 0.000471748 | phosphopantothenate--cysteine ligase |
| ZY05719_02495 | 1.6208721 | 0.000601049 | YdbC family protein |
| ZY05719_08525 | 1.597005272 | 0.000684291 | acetyl-CoA carboxylase carboxyl transferase subunit alpha |
| ZY05719_05585 | 1.587101785 | 0.002901837 | sugar kinase |
| ZY05719_01925 | 1.584208596 | 0.000899785 | endonuclease/exonuclease/phosphatase family protein |
| ZY05719_00630 | 1.583495616 | 0.000775623 | tRNA-Tyr |
| ZY05719_00170 | 1.578796753 | 0.00080303 | tRNA-Phe |
| ZY05719_03930 | 1.53879502 | 0.001054914 | GMP reductase |
| ZY05719_08555 | 1.519356891 | 0.001204917 | 3-oxoacyl-[acyl-carrier-protein] reductase |
| ZY05719_00180 | 1.499803775 | 0.001432335 | tRNA-Ser |
| ZY05719_08530 | 1.466776964 | 0.001746975 | acetyl-CoA carboxylase, carboxyltransferase subunit beta |
| ZY05719_00175 | 1.461454371 | 0.001849509 | tRNA-Ile |
| ZY05719_06515 | 1.454931647 | 0.001900287 | amino acid ABC transporter permease |
| ZY05719_07835 | 1.452297904 | 0.003365655 | IS110 family transposase |
| ZY05719_06510 | 1.423521655 | 0.002359556 | amino acid ABC transporter permease |
| ZY05719_06780 | 1.397216535 | 0.002939488 | tRNA-Ser |
| ZY05719_02490 | 1.362946671 | 0.003573537 | hypothetical protein |
| ZY05719_08310 | -1.385973916 | 0.00311153 | DUF3278 domain-containing protein |
| ZY05719_09160 | -1.401298548 | 0.002826665 | PTS transporter subunit EIIC |
| ZY05719_08315 | -1.437952095 | 0.002344335 | hypothetical protein |
| ZY05719_03045 | -1.512788753 | 0.001275691 | ornithine carbamoyl transferase |
| ZY05719_05440 | -1.519143355 | 0.001560429 | DUF1700 domain-containing protein |
| ZY05719_09825 | -1.538103361 | 0.002979514 | PTS lactose/cellobiose transporter subunit IIA |
| ZY05719_05905 | -1.610235803 | 0.000706383 | DUF3267 domain-containing protein |
| ZY05719_05900 | -1.62837213 | 0.000744919 | helix-turn-helix transcriptional regulator |
| ZY05719_04915 | -1.631606422 | 0.000571197 | glucose-1-phosphate adenylyltransferase subunit GlgD |
| ZY05719_00960 | -1.638129481 | 0.00090199 | carbohydrate ABC transporter permease |
| ZY05719_01420 | -1.641621186 | 0.000498008 | bifunctional acetaldehyde-CoA/alcohol dehydrogenase |
| ZY05719_00945 | -1.650431059 | 0.000461131 | AraC family transcriptional regulator |
| ZY05719_01785 | -1.677170686 | 0.000381735 | LacI family DNA-binding transcriptional regulator |
| ZY05719_04340 | -1.685208094 | 0.000361647 | HAD family hydrolase |
| ZY05719_09100 | -1.687740273 | 0.000552274 | GH92 family glycosyl hydrolase |
| ZY05719_05840 | -1.692174177 | 0.000710644 | glycoside hydrolase family 88 protein |
| ZY05719_09165 | -1.69920089 | 0.000320331 | N-acetylmuramic acid 6-phosphate etherase |
| ZY05719_05895 | -1.706618635 | 0.000368523 | DUF3169 family protein |
| ZY05719_03260 | -1.824684088 | 0.000123997 | cation transporter |
| ZY05719_05915 | -1.916961026 | 0.00010828 | hypothetical protein |
| ZY05719_01510 | -1.995712155 | 2.92E-05 | nucleotide exchange factor GrpE |
| ZY05719_03960 | -1.997502938 | 2.81E-05 | 1-phosphofructokinase |
| ZY05719_02120 | -2.035320252 | 1.97E-05 | ribosome-associated translation inhibitor RaiA |
| ZY05719_02220 | -2.042811559 | 2.48E-05 | beta-galactosidase family protein |
| ZY05719_09865 | -2.044537463 | 1.86E-05 | metal ABC transporter permease |
| ZY05719_03055 | -2.060803666 | 1.69E-05 | YfcC family protein |
| ZY05719_01795 | -2.096258635 | 1.23E-05 | UDP-glucose--hexose-1-phosphate uridylyltransferase |
| ZY05719_03040 | -2.186733152 | 5.21E-06 | GNAT family N-acetyltransferase |
| ZY05719_09870 | -2.187909527 | 5.14E-06 | iron chelate uptake ABC transporter family permease subunit |
| ZY05719_01415 | -2.286832953 | 2.11E-06 | alcohol dehydrogenase AdhP |
| ZY05719_03955 | -2.379955889 | 8.51E-07 | DeoR/GlpR family DNA-binding transcription regulator |
| ZY05719_01880 | -2.386839286 | 1.34E-06 | dynamin family protein |
| ZY05719_09880 | -2.401405233 | 6.82E-07 | zinc ABC transporter substrate-binding protein |
| ZY05719_06395 | -2.454925359 | 1.36E-06 | ABC transporter permease subunit |
| ZY05719_09875 | -2.516914152 | 2.19E-07 | metal ABC transporter ATP-binding protein |
| ZY05719_09280 | -2.529300552 | 1.91E-07 | dihydroxyacetone kinase subunit DhaK |
| ZY05719_03465 | -2.61572445 | 3.96E-07 | glycerol dehydrogenase |
| ZY05719_09170 | -2.631324667 | 1.34E-07 | MurR/RpiR family transcriptional regulator |
| ZY05719_07400 | -2.700756709 | 2.67E-07 | alpha-galactosidase |
| ZY05719_09035 | -2.870884277 | 5.55E-09 | sn-glycerol-3-phosphate ABC transporter ATP-binding protein UgpC |
| ZY05719_09835 | -2.926311771 | 4.70E-09 | PRD domain-containing protein |
| ZY05719_03050 | -3.028798037 | 1.37E-09 | carbamate kinase |
| ZY05719_04910 | -3.100313786 | 4.67E-10 | glucose-1-phosphate adenylyltransferase |
| ZY05719_00955 | -3.101191581 | 2.12E-09 | sugar ABC transporter permease |
| ZY05719_01790 | -3.320983569 | 4.78E-11 | galactokinase |
| ZY05719_03035 | -3.433818157 | 1.02E-11 | arginine deiminase |
| ZY05719_00950 | -3.490179018 | 6.88E-12 | sugar ABC transporter substrate-binding protein |
| ZY05719_09285 | -3.492583238 | 5.33E-12 | dihydroxyacetone kinase subunit DhaL |
| ZY05719_03085 | -4.035864518 | 7.57E-15 | glucosamine-6-phosphate deaminase |
| ZY05719_09290 | -4.194686039 | 1.10E-15 | dihydroxyacetone kinase phosphoryl donor subunit DhaM |
| ZY05719_09295 | -4.698575473 | 1.83E-18 | MIP/aquaporin family protein |
