# Supplementary material for: STK-mediated FadR phosphorylation regulates the acid resistance and virulence of Streptococcus suis
Source: PLoS Pathog. 2025 Sep 25;21(9):e1013534. doi: 10.1371/journal.ppat.1013534 (PMC12463286; doi:10.1371/journal.ppat.1013534)
Supplement: S4 Table — (DOCX) [file ppat.1013534.s018.docx]

**S4 Table.** Strains and plasmids used in this study.

| **Name** | **Characteristics** | **Source** |
| --- | --- | --- |
| **Strains** | | |
| ZY05719 | Isolated from a diseased pig in Sichuan, China | Lab stocks |
| Δ*fadR* | Isogenic *fadR* mutant of ZY05719 | This study |
| FadR-T230A | Point-mutation strain of ZY05719 | This study |
| FadR-T230E | Point-mutation strain of ZY05719 | This study |
| FadR-T230E-C*adi* | Restoring *adi* expression in FadR-T230E | This study |
| Δ*adi* | Isogenic *adi* mutant of ZY05719 | This study |
| C*ΔfadR-flag* | Complemented strain of ZY05719 *ΔfadR* with flag | This study |
| ADI^Imp^ | ZY05719 containing *adi* with *impdh* promoter | This study |
| ADI^Eno^ | ZY05719 containing *adi* with *enolase* promoter | This study |
| *E. coli*DH5α | Cloing host for maintaining the recombinant plasmids | Lab stocks |
| *E. coli* BL21 | The expression host of recombinant proteins | Lab stocks |
| **Plasmids** | | |
| pSET4s | thermosensitive suicide vector; Spc^r^ | Lab stocks |
| pSET2 | *E. Coli*-*S. suis* shuttle vector; Spc^r^ | Lab stocks |
| pET28a | Prokaryotic expression | Lab stocks |
| pGEX4T-1 | Prokaryotic expression | Lab stocks |
| pET28a-*fadR* | Cloning expression of recombinant GlmM | This study |
| pET28a-*fadR*-T230A | Cloning expression of recombinant T230A | This study |
| pET28a-*adi* | Cloning expression of recombinant ADI | This study |
| pGEX4T-1-*nstk* | Cloning expression of recombinant nSTK | This study |
| pSET4s-*fadR* | Recombinant vector designed to knock out *fadR*, Spc^r^ | This study |
| pSET4s-*fadR*-T230A | Recombinant vector designed to point mutation | This study |
| pSET4s-*fadR*-T230E | Recombinant vector designed to point mutation | This study |
| pSET4s-*adi* | Recombinant vector designed to knock out *adi*, Spc^r^ | This study |
| pSET2-*fadR-flag* | pSET2 containing *fadR-flag*, Spc^r^ | This study |
| pSET2-*adi-imp* | pSET2 containing *adi with impdh* promoter, Spc^r^ | This study |
| pSET2-*adi-eno* | pSET2 containing *adi with enolase* promoter, Spc^r^ | This study |
