# Supplementary material for: STK-mediated FadR phosphorylation regulates the acid resistance and virulence of Streptococcus suis
Source: PLoS Pathog. 2025 Sep 25;21(9):e1013534. doi: 10.1371/journal.ppat.1013534 (PMC12463286; doi:10.1371/journal.ppat.1013534)
Supplement: S5 Table — (DOCX) [file ppat.1013534.s019.docx]

**S5 Table.** Primer used in this study.

| **Primers** | **Sequence (5’-3’)** |
| --- | --- |
| **For strains construction** | |
| *ΔfadR*-F1 | GAGCTCGGTACCCGGGGATCCAGGACATCCCGCCTGACTAGC |
| *ΔfadR*-R1 | TGTATTGGAGGACCAAGCACAAATAAAAAATAGACAGCCC |
| *ΔfadR*-F2 | GGGCTGTCTATTTTTTATTTGTGCTTGGTCCTCCAATACA |
| *ΔfadR*-R2 | CAGGTCGACTCTAGAGGATCCCGCCTGATAAGGATGAGGTTATG |
| *Δstk*-F1 | CAGGTCGACTCTAGAGGATCCGCTGAAAACCAAAAGATTCATGAA |
| *Δstk*-R1 | CTCCCCAACCTTATTCATCTTACCTAGCCTCCTCCGTAAT |
| *Δstk*-F2 | ATTACGGAGGAGGCTAGGTAAGATGAATAAGGTTGGGGAG |
| *Δstk*-R2 | AAAACGACGGCCAGTGAATTCGCCTCATTGGCTACCACATCA |
| FadR-F | GAGCTCGGTACCCGGGGATCCAGGACATCCCGCCTGACTAGC |
| FadR-R | CAGGTCGACTCTAGAGGATCCCGCCTGATAAGGATGAGGTTATG |
| FadR-T230A-F | TAAAGATATGGCATTGCATGGTCTCAACGATCATAAATAG |
| FadR-T230A-R | GACCATGCAATGCCATATCTTTATCGAACCATTCGCTTAA |
| FadR-T230E-F | TAAAGATATGGAATTGCATGGTCTCAACGATCATAAATAG |
| FadR-T230E-R | GACCATGCAATTCCATATCTTTATCGAACCATTCGCTTAA |
| *Δadi*-F1 | GAGCTCGGTACCCGGGGATCCTCAGATCGAGTCGTTCAAGCC |
| *Δadi*-R1 | TGTTCGTTCTATACTTGGCACTGTCCTCCTTGTAGATTTA |
| *Δadi* -F2 | TAAATCTACAAGGAGGACAGTGCCAAGTATAGAACGAACA |
| *Δadi* -R2 | CAGGTCGACTCTAGAGGATCCTTTCTCTGCAAGGAAATGTCTACCT |
| C*ΔfadR*-*flag*-F | ACATGCATGCGGGCTCCGTCAAGAGTTGGA |
| C*ΔfadR*-*flag*-R | CGGAATTCTTACTTGTCATCGTCGTCCTTGTAGTCTTTATGATCGTTGAGACCAT |
| C-*adi*-*imp*-F1 | GAGCTCGGTACCCGGGGATCCATGGAGGCAGGACAGGTATTTTT |
| C-*adi*-*imp*-R1 | ATTGGATGGTTTGACATGATGTTCTTTCCTTTCTTTTGGG |
| C-*adi*-*imp*-F2 | CCCAAAAGAAAGGAAAGAACATCATGTCAAACCATCCAAT |
| C-*adi*-*imp*-R2 | CAGGTCGACTCTAGAGGATCCTTAGATGTCTTCACGTTCAAACGG |
| C-*adi*-*eno*-F1 | AAAACGACGGCCAGTGAATTCTGTTTCGCCAGAGGCTTTCT |
| C-*adi*-*eno*-R1 | ATTGGATGGTTTGACATGATTATATTACTCTCCTTTGAGT |
| C-*adi*-*eno*-F2 | ACTCAAAGGAGAGTAATATAATCATGTCAAACCATCCAAT |
| C-*adi*-*eno*-R2 | Same as C-*adi*-*imp*-R2 |
| **Prokaryotic expression** | |
| nSTK-F | CGCGGATCCATGATTCAAATCGGTAAGATC |
| nSTK-R | CCGCTCGAGTGTATCAACCTTGTTCCC |
| B-FadR-F | CGGAATTCATGGCGAAACCCTTGGTAGA |
| B-FadR-R | GCGTCGACCTATTTATGATCGTTGAGAC |
| B-FadR-T230A-F | AGATATGGCATTGCATGGTCTCAACGATCA |
| B-FadR-T230A-R | CATGCAATGCCATATCTTTATCGAACCATT |
| B-FadR-T230E-F | GATAAAGATATGGAGTTGCATGGTCTCAACGATCATAAAT |
| B-FadR-T230E-R | GAGACCATGCAACTCCATATCTTTATCGAACCATTCGCTT |
| ADI-F | CAGCAAATGGGTCGCGGATCCATCATGTCAAACCATCCAATTCAT |
| ADI-R | ACGGAGCTCGAATTCGGATCCTTAGATGTCTTCACGTTCAAACGG |
| GAPDH-F (P) | CGGGATCCATGGTAGTTAAAGTTGGTAT |
| GAPDH-R (P) | CGGAATTCTTATTTAGCGATTTTTGCGA |
| **For RT-qPCR** | |
| FadR-F (qPCR) | GTCGCTCAGTTTGCCTCGGA |
| FadR-R (qPCR) | TCCCACTCATCTCAGCCAGCA |
| ADI-F (qPCR) | ATTGCACCAGGTGTGGTAGT |
| ADI-R (qPCR) | ACGTTCAAACGGCATTGACA |
| GAPDH-F (qPCR) | TAAACTTGACGGTGCTGCAC |
| GAPDH-R (qPCR) | CCAATTGCTCGCCATCAACT |
| **For EMSA or ChIP** | |
| Promoter-*adi-*F | AGATATTGATTATTTCTTAG |
| Promoter-*adi*-R | CTGTCCTCCTTGTAGATTTA |
| Promoter-*fadR-*F | GTGCTTGGTCCTCCAATACA |
| Promoter-*fadR*-R | ATGTGGGATATTCCTTATAC |
| 16S-F | GTAACCTGCCTCATAGCG |
| 16S-R | ATTGCCGAAGATTCCCTA |
